# Overcoming Antigenic Diversity by Enhancing the Immunogenicity of Conserved Epitopes on the Malaria Vaccine Candidate Apical Membrane Antigen-1

**DOI:** 10.1371/journal.ppat.1003840

**Published:** 2013-12-26

**Authors:** Sheetij Dutta, Lisa S. Dlugosz, Damien R. Drew, Xiopeng Ge, Diouf Ababacar, Yazmin I. Rovira, J. Kathleen Moch, Meng Shi, Carole A. Long, Michael Foley, James G. Beeson, Robin F. Anders, Kazutoyo Miura, J. David Haynes, Adrian H. Batchelor

**Affiliations:** 1 Walter Reed Army Institute of Research, Silver Spring, Maryland, United States of America; 2 Burnet Institute, Melbourne, Victoria, Australia; 3 Department of Biochemistry, La Trobe University, Victoria, Australia; 4 Laboratory of Malaria and Vector Research, National Institutes of Health, Rockville, Maryland, United States of America; MRC National Institute for Medical Research, United Kingdom

## Abstract

Malaria vaccine candidate Apical Membrane Antigen-1 (AMA1) induces protection, but only against parasite strains that are closely related to the vaccine. Overcoming the AMA1 diversity problem will require an understanding of the structural basis of cross-strain invasion inhibition. A vaccine containing four diverse allelic proteins 3D7, FVO, HB3 and W2mef (AMA1 Quadvax or QV) elicited polyclonal rabbit antibodies that similarly inhibited the invasion of four vaccine and 22 non-vaccine strains of *P. falciparum*. Comparing polyclonal anti-QV with antibodies against a strain-specific, monovalent, 3D7 AMA1 vaccine revealed that QV induced higher levels of broadly inhibitory antibodies which were associated with increased conserved face and domain-3 responses and reduced domain-2 response. Inhibitory monoclonal antibodies (mAb) raised against the QV reacted with a novel cross-reactive epitope at the rim of the hydrophobic trough on domain-1; this epitope mapped to the conserved face of AMA1 and it encompassed the 1e-loop. MAbs binding to the 1e-loop region (1B10, 4E8 and 4E11) were ∼10-fold more potent than previously characterized AMA1-inhibitory mAbs and a mode of action of these 1e-loop mAbs was the inhibition of AMA1 binding to its ligand RON2. Unlike the epitope of a previously characterized 3D7-specific mAb, 1F9, the 1e-loop inhibitory epitope was partially conserved across strains. Another novel mAb, 1E10, which bound to domain-3, was broadly inhibitory and it blocked the proteolytic processing of AMA1. By itself mAb 1E10 was weakly inhibitory but it synergized with a previously characterized, strain-transcending mAb, 4G2, which binds close to the hydrophobic trough on the conserved face and inhibits RON2 binding to AMA1. Novel inhibition susceptible regions and epitopes, identified here, can form the basis for improving the antigenic breadth and inhibitory response of AMA1 vaccines. Vaccination with a few diverse antigenic proteins could provide universal coverage by redirecting the immune response towards conserved epitopes.

## Author Summary

Numerous reports of vaccine failure are attributed to a mismatch between the genotype of the vaccine and the circulating target strains. This observation is congruent to the view that polyvalent vaccines protect broadly by inducing a multitude of type-specific antibodies. Polyvalent vaccines that can overcome antigenic diversity by refocusing antibody responses towards conserved functional epitopes are highly desirable. Development of an Apical Membrane Antigen-1 (AMA1) malaria vaccine has been impeded by extreme antigenic diversity in the field. We present here a solution to the AMA1 diversity problem. Antibodies against a mixture of only four naturally occurring AMA1 allelic proteins “Quadvax” inhibited invasion of red blood cells by a diverse panel of malaria parasites that represented the global diversity of AMA1 in the field. Competition experiments suggested that in addition to improving the diversity of strain-specific antibodies, the mechanism of broadened inhibition involved an increase in responses against conserved inhibitory epitopes. Monoclonal antibodies against the Quadvax inhibited invasion either by blocking the binding of AMA1 to its receptor RON2 or by blocking a crucial proteolytic processing event. Some mixtures of these antibodies were much more effective than expected and were shown to act synergistically. Together these two classes of functional invasion inhibitory epitopes can be targeted to engineer a more potent AMA1 vaccine.

## Introduction

Despite the availability of effective drugs mortality caused by malaria remains a global health and economic concern [1], and drug resistance to front-line antimalarials is increasing. A vaccine that either prevents the disease or can reduce the parasite burden is urgently needed to reduce mortality and morbidity of infants and young children living in many of the world's poorest countries. Human anti-malarial antibodies can inhibit invasion and some studies suggest that growth inhibitory activity is associated with decreased risk of *Plasmodium falciparum* infection [2]. Antigens that induce invasion inhibitory antibodies are therefore primary candidates for the development of a vaccine that targets parasite blood stages [3], [4]. One such vaccine candidate is Apical Membrane Antigen-1 (AMA1) [5]. Anti-AMA1 antibodies inhibit the invasion of merozoites into red blood cells [6] and this inhibitory activity correlates with protection in non-human primate malaria models [7], [8]. Inhibitory AMA-1 antibodies are also acquired by humans exposed to *P. falciparum* infection [9], and antibodies to AMA1 are associated with protection from symptomatic malaria [10], [11]. The positive selection of polymorphisms that map to the epitopes of inhibitory antibodies is further evidence that such antibodies have a protective role [12].

*In vitro* growth inhibitory activity and AMA1-induced protection in animal models and in humans are highly strain-dependent [9], [13]–[16]. When evaluated in a Phase 2b clinical trial in Mali, a monovalent 3D7 AMA1 vaccine [17] formulated in an oil-containing adjuvant AS02 showed significant efficacy, but only against vaccine-like strains [15]. Although disappointing, this result was not surprising given the parasite diversity at the test site [18]. Vaccinating with a yeast-derived bivalent mixture of 3D7 and FVO alleles (AMA1-C1) did not enhance the inhibition against non-vaccine strains [19] and this bivalent vaccine adjuvanted in Alum did not protect in a Phase 2b trial [20].

Synthesized as an 83 kDa trans-membrane protein, native AMA1 undergoes maturation to a 66 kDa form, which then translocates to the merozoite surface [21]. During the invasion process, AMA1 undergoes proteolytic shedding to yield 48 and 44 kDa soluble forms [22] [23]. Once on the merozoite surface, the 66 kDa AMA1 form interacts with the parasite RON proteins, which are integrated into the host cell membrane [24]. A portion of the RON2 protein binds within a trough of exposed hydrophobic residues of AMA1 domain-1, and this interaction is thought to be necessary for triggering formation of an actinomyosin-associated moving junction that drives host cell invasion [25], [26]. The understanding of the biological role of AMA1 during invasion has prompted researchers to target the AMA1-RON2 interaction for vaccine development [27]. However polymorphisms located on loops that surround the hydrophobic trough are the major antigenic escape residues of AMA1 [28], and invasion inhibitory monoclonal antibodies (mAbs) like 1F9, that map to the rim of hydrophobic trough are highly strain-specific [29]. The crystal structure of AMA1 has revealed that AMA1 polymorphisms cluster on one side of the AMA1 molecule and mAb 4G2, which binds to the opposite “conserved face”, is broadly inhibitory [30]–[33]. Although the epitope for mAb 4G2 offers the potential for rational vaccine design, this epitope is not easily accessible and intact mAb 4G2 is about 40 times less inhibitory than polyclonal AMA1 antibodies [34]. Thus a major problem with applying structural approaches to improve AMA1 vaccines has been the lack of well characterized mAbs that are cross-reactive and whose growth inhibitory activity approaches that of polyclonal AMA1 antibodies.

To be successful, vaccines against pathogens that exhibit antigenic diversity require the inclusion of multiple components. The polio vaccine contains all three circulating serotypes, the influenza vaccine contains three seasonally prevalent serotypes whose antibodies are functionally non cross-reactive and human papilloma virus vaccine contains the four most pathogenic types. However, extreme diversity in AMA1 with over 200 prevailing haplotypes has precluded the inclusion of all AMA1 strains into a multivalent vaccine [18], [35] and several important questions need to be answered before developing the next generation of AMA1 vaccines. For example, if pan-inhibition requires the presence of a multitude of strain-specific antibodies, then most serotypes will have to be present in the vaccine. On the other hand, if the immunogen can induce high levels of broadly inhibitory antibodies, all serotypes need not be present.

A sequence diversity based approach rationally classified AMA1 sequences using a clustering algorithm and suggested that no less than 6 populations would be required in a vaccine [36]. However, Miura *et al.* showed that these populations do not clearly explain the patterns of cross-strain inhibition in growth or invasion inhibition assays (GIA) and all six populations may not be necessary for a multivalent vaccine to overcome antigenic diversity [37]. Remarque and colleagues have proposed to display majority of AMA1 polymorphisms on artificially designed diversity covering (DiCo) proteins. Vaccination of rabbits and monkeys with mixtures of DiCo proteins elicited broadly cross-reactive antibodies that inhibited several laboratory strains [38], [39]. The DiCo approach has enabled the concentration of 97% of AMA1 polymorphisms on only three proteins and it assumes that all polymorphisms are equally important. This assumption however has not been supported by the literature [28], [37], [40]. DiCo mix vaccine also remains to be evaluated against a broad array of laboratory and recent field isolates. A mixture of two AMA1 alleles, 3D7 and FVO, did not increase non-vaccine strain inhibition, but adding a third allele, HB3, achieved some level of strain-broadening [41], [42]. Further improvements in cross-strain inhibition were reported using mixtures of 4/5 and 6 allele vaccines [37].

In view of the failure of AMA1 vaccines to induce broad protection against malaria, understanding of the structural basis of cross-strain invasion inhibition is required to make a case for developing second generation AMA1 vaccines. The aim of this study was to understand the structural basis of cross-strain invasion inhibition by mixed allele vaccine antibodies. To induce broadly inhibitory antibodies we mixed four diverse AMA1 allelic proteins to constitute the Quadvax or QV. The four allelic components of the QV (3D7, FVO, HB3, W2mef) show limited cross-inhibition in a GIA [9], [14], [19] and a four-way pool of their individual antibodies can inhibit a wide variety of non-vaccine strains [43]. The breadth of inhibitory response of QV, was compared to a bivalent, two trivalent and four monovalent vaccines against a panel of laboratory and recently culture adapted isolates. Broadly inhibitory monoclonal antibodies (mAbs) generated against QV were mapped and assayed for biological activities and mAbs and chimeric proteins were used to discern differences between multi-allelic and mono-allelic AMA1 vaccine responses. Understanding the molecular basis of strain broadening would allow for the rational development and testing of a second generation AMA1 vaccine.

## Results

### Anti-QV inhibited vaccine and non-vaccine strains similarly

**Figure 1:**
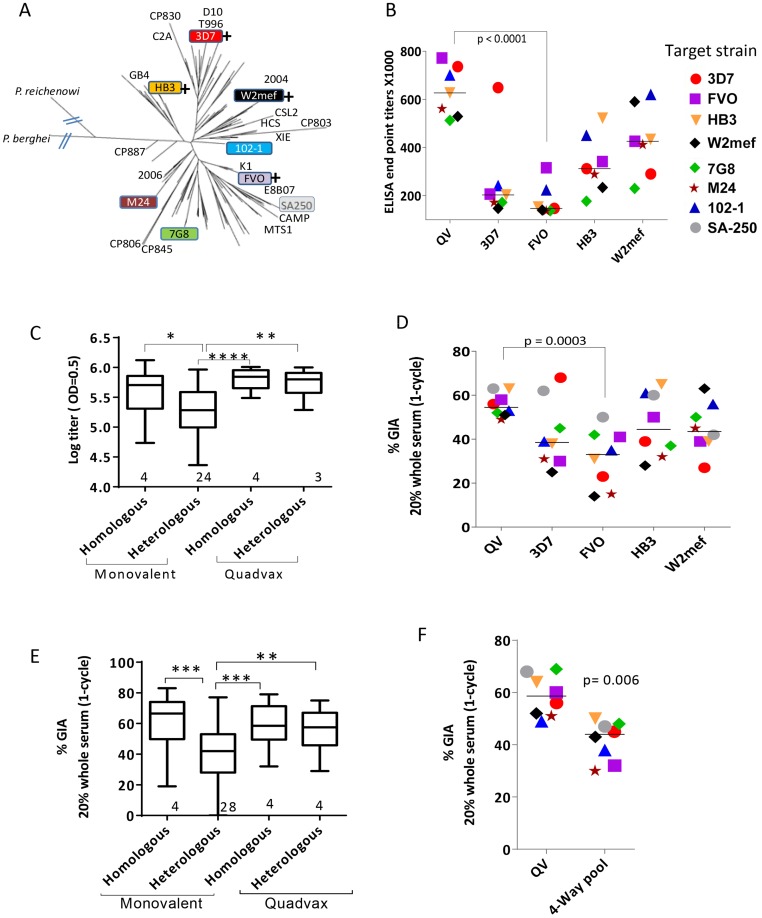
AMA1 sequence diversity and allelic coverage of four monovalent vaccines as compared to the QV. (**A**) A dendrogram constructed with full-length AMA1 sequences from the 26 target strains tested in GIA and 175 field strain sequences obtained from Genbank (Table S1). *P. berghei* (rodent) and *P. reichenowi* (chimpanzee) sequences were also included. (+) indicates that this allelic AMA1 protein was included in QV. The colored boxes were the 8 target strains used in the WRAIR GIA. (**B**) ELISA titers (×1000) for the five vaccine groups tested against 7 allelic proteins. Symbols are mean of three rabbits and lines are median titer across strains. (**C**) Box-and-whiskers plot using individual rabbit ELISA data grouped on the basis of whether the coat antigen-antisera combinations were homologous or heterologous and whether monovalent or QV rabbits were tested. The number under each box represents the total number of protein-antisera combinations included. (*) indicates, p<0.01, (**) p<0.001, (***) p<0.0001 and (****) p<0.0001 for ANOVA followed by Tukey's multiple comparisons test. (**D**) One-cycle GIA of the five vaccine groups against four non-vaccine and four vaccine strains using 1∶5 whole serum dilution. Symbols in Fig. 1B and 1D and 1F are matched, except strain SA250 that was only tested in the GIA. Each symbol is mean of three rabbits tested in two experiments and lines are median inhibition across strains. (**E**) GIA data from individual rabbits from three experiments grouped similar to the ELISA data, except the groups were made based on homologous and heterologous parasite-antisera combinations. (**F**) GIA activity of pooled QV sera was compared to a pool of the highest titer rabbit sera in the four monovalent vaccine groups 3D7+FVO+HB3+W2mef (4-Way pool). Lines are median inhibition across 8 target parasite strains; representative of 2 experiments is shown.

Groups of three rabbits were immunized with monovalent 3D7, FVO, HB3 and W2mef AMA1 vaccines or an equivalent total antigen dose of a mixture of all four allelic proteins (QV). To determine the antigenic breadth of the induced antibodies, individual rabbit sera were analyzed by ELISA against recombinant proteins corresponding to seven diverse AMA1 alleles (Fig. 1A). The QV antisera showed a high degree of cross-reactivity (>500,000 mean group titer against all 7 allelic proteins; Fig. 1B) whereas the monovalent vaccine antisera showed the typical strain-specificity of AMA1 antibodies. Mean log_10_ ELISA titers of the four monovalent vaccines, tested against their respective homologous target strains, were not different from those induced by QV (MANOVA followed by Dunnett's test all p values >0.1). When the monovalent vaccine-induced titers were grouped together, the combined mean homologous strain titer was higher than the heterologous strain titer (ANOVA, followed by Tukey's test; Fig. 1C). In contrast, the QV group showed no difference in homologous and heterologous AMA1 titers. In a GIA that measured parasitemia after one invasion cycle, using a flow-cytometric method (WRAIR GIA) [44], anti-QV showed similarly high levels of inhibition of homologous and four heterologous parasite strains (>49% inhibition at 1∶5 whole serum dilution; Fig. 1D), while the GIA activity of the monovalent vaccines was dependent on the test strain. Homologous strain inhibitions of the QV group were similar to the homologous inhibitions induced by the monovalent vaccines (Dunnett's test p values>0.2). Similar to the grouped ELISA analysis, the combined mean homologous inhibition by the monovalent vaccine antisera was higher than heterologous inhibition, but no such difference for anti-QV was observed (Fig. 1E).

A plot of ‘vaccine to target’ sequence distance showed an inverse correlation between sequence distance and invasion inhibition, for 3D7, HB3 and W2mef vaccines (linear regression, p<0.0001, 0.040 and 0.0006, respectively), which confirmed that GIA was detecting antigenic escape *in vitro* (Fig. S1). In contrast, the slope for QV and FVO regression lines (drop in GIA per polymorphism) was relatively flat and the correlations were non-significant. Although sequence distance was not predictive of immune escape from anti-FVO, the antibody response and GIA activity induced by FVO AMA1 were significantly lower than anti-QV (t-test, p<0.0001 and 0.0003 respectively; Fig. 1B and 1D).

Using a 4-Way pool of antibodies against the monovalent vaccines, given separately to rabbits using Freund's complete adjuvant, Drew *et al.* have shown broad inhibitory coverage against diverse strains [43]. Hence we compared the activity of pooled QV rabbit sera to a 4-Way pool of sera from the four highest titer monovalent vaccine group rabbits (anti-3D7+FVO+HB3+W2mef) (Fig. 1F). It is notable that GIA activity across strains for the QV pool was higher than the 4-Way pool (t-test, p = 0.006). This data along with the higher heterologous coverage judged by GIA and ELISA (Fig. 1C and 1E), indicates that anti-QV did not merely represent the sum of strain-specific antibodies and in contrast to the dilution of inhibitory activity observed upon mixing monovalent vaccine antisera the mixed allele vaccine maintained a high level of inhibition across strains.

### QV induced higher levels of broadly inhibitory antibodies

**Figure 2:**
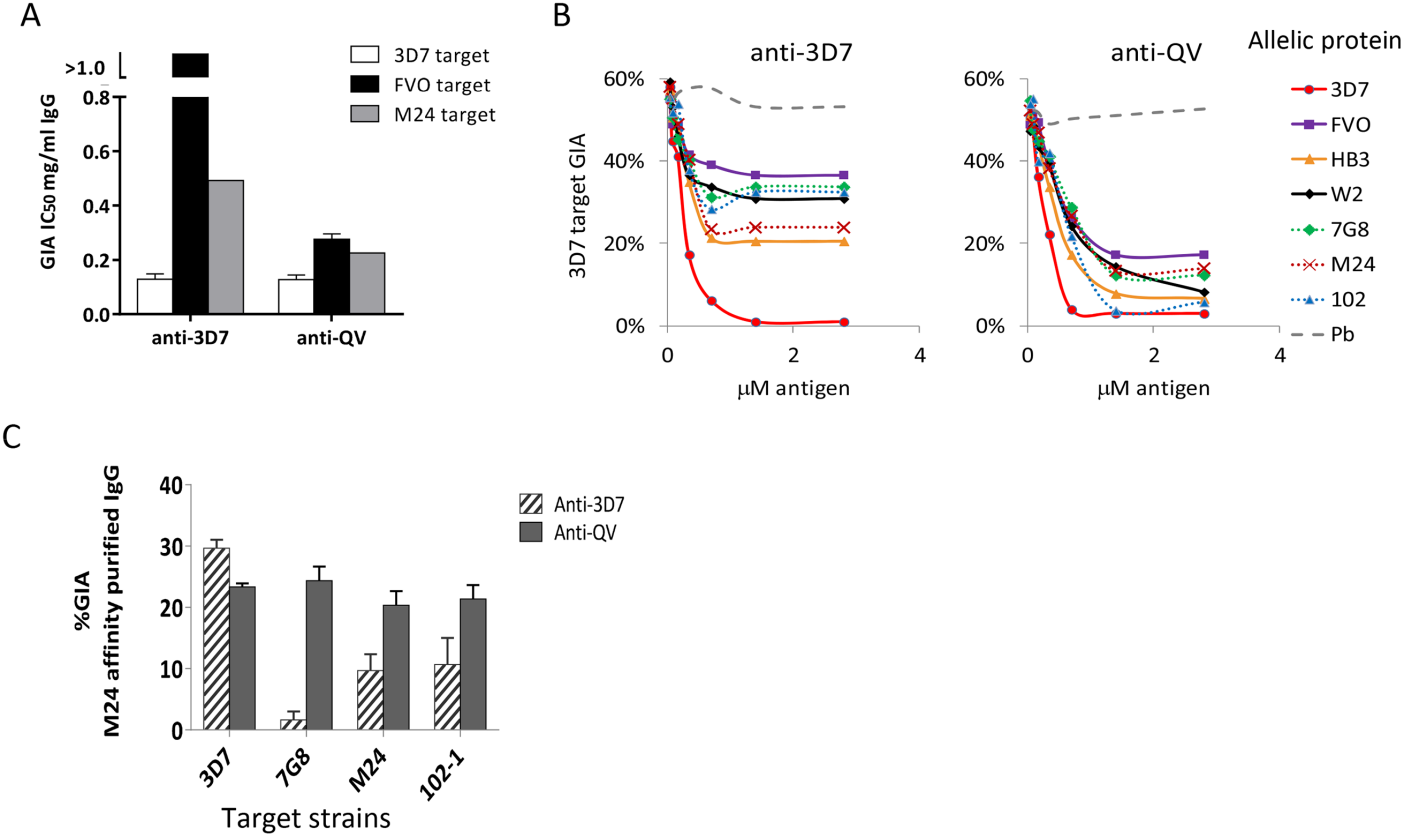
Quantification of broadly inhibitory antibodies. (**A**) IC_50_ values against three target strains for the monovalent anti-3D7 and anti-QV IgG, affinity purified from pools of 3 rabbit sera, using a 3D7 AMA1 column (GIA in Fig. S2). (**B**) GIA reversal comparing the ability of AMA1 allelic proteins to reverse the inhibition of 3D7 parasite invasion by pools of anti-QV or anti-3D7 sera. *P. berghei* AMA1 was used as the negative control (Pb). The data is representative of 2 experiments. (**C**) GIA of pooled anti-3D7 and anti-QV IgG (3 rabbits each) that was bound and eluted from a M24 AMA1 affinity column and tested at 0.15 mg/ml against 3D7 or three non-vaccine parasite strains (7G8, M24 and 102-1). Mean+s.e.m from 3 experiments is shown.

Sera from the three QV vaccinated rabbits were pooled to isolate antigen-specific antibodies over a 3D7 AMA1 affinity column. As a control, antibodies from a pool of three monovalent 3D7 AMA1 vaccinated rabbits were affinity purified in parallel. More than 3.5 times as much anti-3D7 IgG was required for 50% inhibition (IC_50_) of heterologous strains as was required for 50% inhibition of 3D7 parasites (Fig. 2A and Fig. S2). In contrast, the anti-QV IC_50_ against 3D7, FVO and M24 strains were similarly low. Notably, the flow-through fraction of anti-QV (unbound antibodies) still showed some level of inhibition of FVO and M24 parasites, while the flow-through of anti-3D7 did not (Fig. S2).

Since anti-3D7 and anti-QV sera showed similar inhibitory activities against the 3D7 target strain, we next determined if both antisera targeted a similar proportion of strain-specific and cross-reactive epitopes. A serial dilution of soluble antigens from seven diverse AMA1 strains, were used to selectively deplete cross-reactive antibodies from serum pools of 3 rabbits in a GIA against 3D7 parasites (Fig. 2B). Vaccine strain (solid lines) and non-vaccine strain (dotted lines) AMA1 proteins similarly reversed anti-QV mediated inhibition, whereas the anti-3D7 inhibition was completely reversible only by the homologous antigen. At saturating antigen concentrations, the three non-vaccine allelic proteins 7G8, M24, and 102-1 were significantly less effective at reversing the inhibition of anti-3D7 antibodies than they were at reversing the inhibition of anti-QV antibodies (average reversal 52% *vs.* 79%; t-test p<0.0001) (Fig. S3).

We also directly compared the relative inhibitory activities of the cross-reactive antibody fraction of anti-3D7 and anti-QV IgG affinity purified from pooled serum of 3 rabbits over a non-vaccine strain M24 AMA1 column (Fig. 2C). The net amount of anti-3D7 that bound to the M24 AMA1 column was lower (8% by weight) than anti-QV (51%) and, despite affinity purification, the cross-reactive fraction of anti-3D7 still showed strain-specific inhibition (highest response against 3D7) which was significantly higher than its inhibition of 7G8, M24 and 102-1 (ANOVA followed by Tukey's test, p = 0.0014, 0.0074 0.0096 respectively). There was no significant difference among the 3D7, 7G8, M24 and 102-1 strains in the level of inhibition by anti-QV IgG (Fig. 2C). These data suggested that, not only did QV induce higher levels of cross-reactive antibodies than the monovalent 3D7 AMA1 vaccine, but a higher proportion of the anti-QV antibodies targeted conserved inhibitory epitopes on the parasite AMA1.

### A combination of four AMA1 variants (QV) may be sufficient to overcome global AMA1 diversity

**Figure 3:**
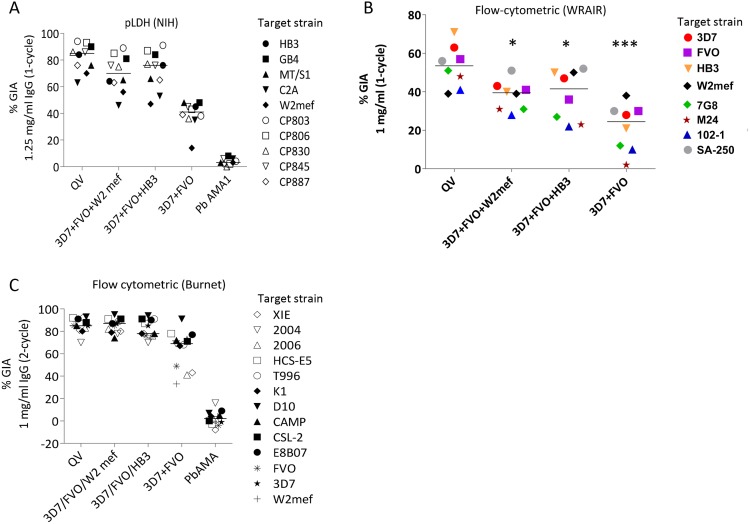
Inhibitory coverage against laboratory and field isolates. (**A**) One-cycle GIA at 1.25 mg/ml total IgG pool from 3 rabbits tested by the NIH pLDH assay. GIA of anti-QV was compared to trivalent and bivalent vaccine groups and antibodies against *P. berghei* AMA1 (PbAMA1) were tested as the negative control. Strains CP803, CP806, CP830, CP845, and CP887 were recent culture adapted Cambodian isolates and HB3, GB4, MT/S1, C2A, W2mef were laboratory strains. Lines are median inhibition across strains. (**B**) One-cycle GIA at 1 mg/ml total IgG pool from 3 rabbits conducted by the WRAIR flow-cytometric method against 8 parasite strains. (*) indicates, p<0.05; (***) p<0.0001 (corrected for multiple comparisons). (**C**) Two-cycle GIA at 1 mg/ml pooled IgG conducted by the Burnet Institute flow-cytometric method. Strains CSL-2, HCS-E5, 2006, 2004, XIE were recently culture adapted field isolates from Africa, Asia and isolates E8B07, CAMP, D10, K1, T996 were laboratory strains [43].

In an independent vaccination experiment, groups of three rabbits were immunized in parallel with 100 µg of QV, or 100 µg mixtures of two (3D7+FVO) or three (3D7+FVO+HB3 and 3D7+FVO+W2mef) allelic proteins. Pooled IgG from each of the four vaccine groups were tested for inhibition of invasion against ten target parasite strains by the National Institutes of Health GIA reference laboratory using a parasite LDH based method following one invasion cycle [45]. The target strains included five recently culture adapted Cambodian isolates (labeled as CP in Fig. 3A). Adding a third allelic protein dramatically improved the cross-strain GIA activity of the bivalent vaccine, and a smaller increase in mean inhibition across strains was observed upon adding the fourth allelic protein to the vaccine, although the mean inhibition across strains for the two trivalent vaccines was not statistically different from the QV. At 2.5 mg/ml total IgG concentration the anti-QV showed uniformly high, >89% inhibition, against all 10 isolates, 8 of which were not included in QV (Fig. S4A). A dose response assay using the pLDH method, determined that a relatively low <0.2 mg/ml total anti-QV IgG resulted in 50% inhibition against the 3D7 target strain (Fig. S4B). When tested for GIA activity against eight *P. falciparum* strains using the WRAIR flow-cytometric assay, inhibition across strains was significantly greater with the anti-QV IgG pool than with IgG induced by either of the two trivalent (ANOVA followed by Dunnett's test, p = 0.033, 0.028) and the bivalent vaccine (p<0.0001) (Fig. 3B). A high level of cross-strain GIA activity with anti-QV IgG was independently verified in assays performed at the Burnet Institute (Melbourne, Australia) using a flow-cytometric assay that measured inhibition over two invasion cycles [46]. An additional ten parasite strains, five of which were recently culture adapted field isolates from south-east Asia and Africa [43], were all found to be highly inhibited by anti-QV and in this more sensitive assay the two trivalent antisera performed similar to the QV (Fig. 3C).

The full-length AMA1 sequences, visualized on a dendrogram against 175 published AMA1 sequences from Asian, South American and African origin (Fig. 1A), showed that the diversity of the 26 target strains, tested by GIA, was representative of the global AMA1 diversity. Although GIA methodologies used by the three labs were different, they all suggested that a combination of three and preferably four QV allelic proteins may be sufficient to provide coverage against global AMA1 diversity .

### Genaration and mapping of monoclonal antibodies against QV

**Figure 4:**
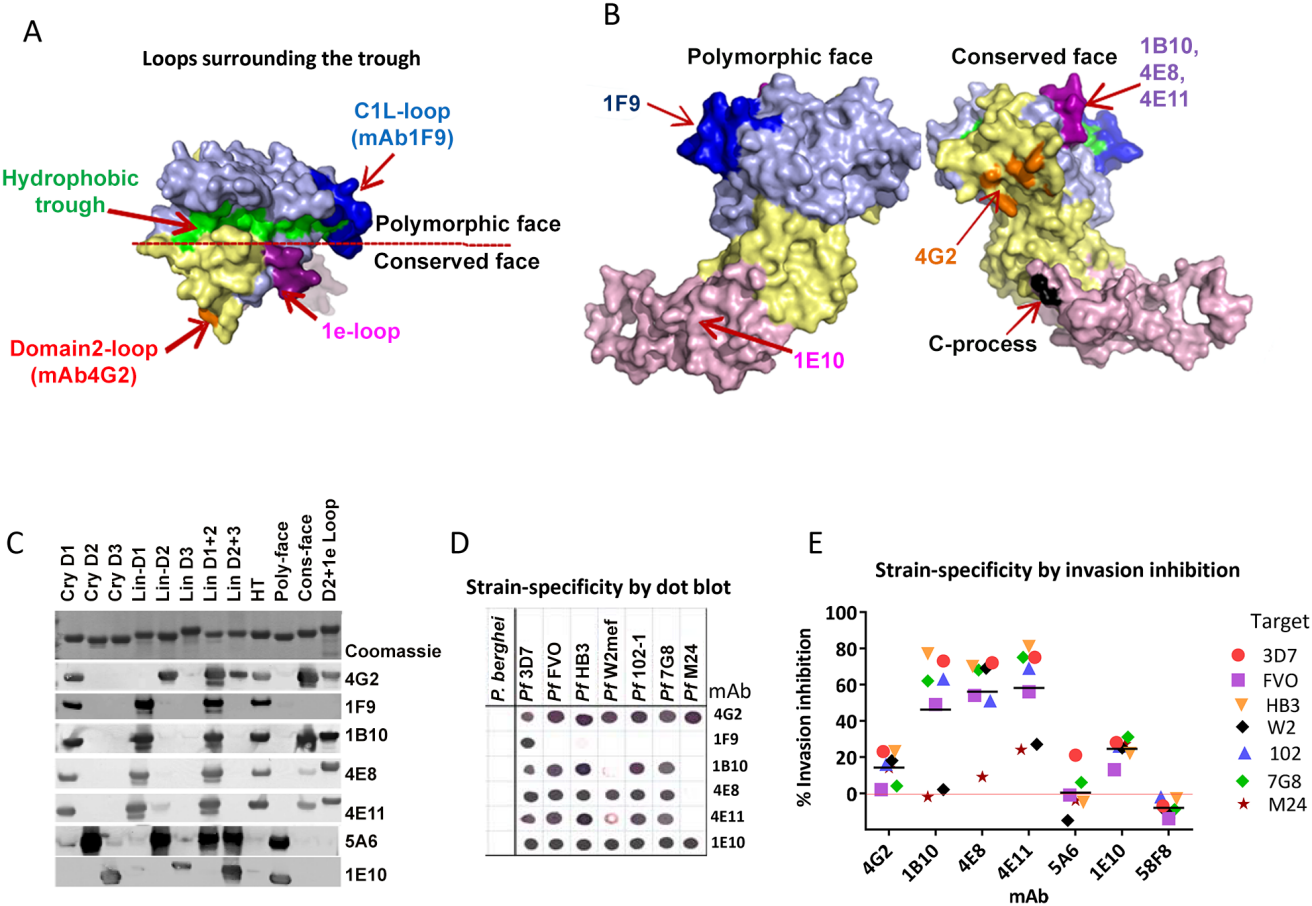
Mapping inhibitory monoclonal antibodies. (**A**) View of the hydrophobic trough and the surrounding loops showing approximate spatial location of mAb epitopes. (**B**) Polymorphic and conserved face of AMA1. Domain-1 residues (light blue); domain-2 (yellow); domain-3 (magenta); C-terminal processing site at residue Thr_517_ (black); mAb 4G2 binding residues (orange); mAb 1F9 epitope centered on the C1L-loop (dark blue); and mAb 1B10, 4E8 and 4E11 epitopes on the 1e-loop (purple). (**C**) Coomassie blue stained and western blot panels showing chimeric proteins (Fig. S5) used to map representative mAbs. (**D**) Dot blot reactivity pattern of inhibitory mAbs against diverse *P. falciparum* AMA1 allelic proteins and *P. berghei* AMA1 control. (**E**) A cross-strain GIA against 7 parasite strains at 1 mg/ml mAb concentration.

To further characterize QV-induced antibodies, a panel of monoclonal antibodies (mAbs) were generated (Table 1 and Fig. 4). Binding domains for the mAbs were assigned by Western blot against a panel of chimeric proteins that displayed *P. falciparum* sequences on a *P. berghei* AMA1 scaffold (Fig. S5 and Fig. 4C). There is 52% sequence identity between *P. falciparum* and *P. berghei* AMA1. This level of identity is similar to that of *P. vivax* AMA1 (58%) which is known to have an identical fold to *P. falciparum* AMA1 [47], and is considerably higher than the identity to *T. gondii* AMA1 (32% identity in domains I and II), known to have an identical fold in the core domain I+II region [48]. Hence, there is precedence for expecting that *P. berghei* and *P. falciparum* AMA1 possess identical folds even though their surfaces are antigenically non-cross-reactive (no cross-reactivity between Pf mAbs and PbAMA1; Fig. 4D). The use of chimeric proteins to map epitopes was necessary because of the conformational nature of AMA1 inhibitory epitopes and attempts to express individual domains often results in structural alterations as was shown by differences in the NMR structure of isolated domain-3 and the structure of domain 3 in context of domain-1 and −2 [33] [49], [50]. Chimeras Cry-D1, Cry-D2, Cry-D3 displayed the contiguous surface regions of 3D7 AMA1 domains-1, 2 and 3 based on the crystal structure (Fig. S5). Chimeras POLY and CONS displayed the polymorphic and conserved face of AMA1; chimera D2+1e displayed the domain-2 loop together with the 1e-loop; and chimera HT displayed the rim of the hydrophobic trough and surrounding loops (Fig. S5) [32]. Also displayed on the chimeras were combinations of the three linear domains of *P. falciparum* AMA1 (Lin-D1, Lin-D2, Lin-D3, LinD1+2 and LinD2+3), as defined by the disulphide bond structure [51]. In addition to Western blot, AMA1 chimeras were useful to generally differentiate the domain/region-specific inhibition patterns of polyclonal sera using a GIA reversal assay (Fig. S5C). Interestingly, all three domains and both the faces of AMA1 had a partial contribution towards invasion inhibition but when combinations of Pf-Pb chimeras encompassing all three domains or both faces of AMA1 were mixed and added, ∼100% reversal of anti-3D7 AMA1-mediated inhibition of 3D7 strain parasites was observed (Fig. S5C).

**Table 1:** Monoclonal antibodies against AMA1.

Binding Location	mAb	GIA 3D7 1 mg/ml	Strain reactivity	Linear chimera	Crystal chimera
D1	1F9* #	17%	1	Lin-D1	Cry-D1
	1B10	65%	5		
	4E8	67%	6		
	4E11	62%	5		
D2 loop	4G2*	22%	7	Lin-D2	Cry-D1
	2B7	−1%	6		
	5B7	−2%	6		
	3D8	−2%	6		
	2C10	−2%	3		
D2	5A6	7%	1	Lin-D2	Cry-D2
	91F	9%	1		
	1F3	10%	1		
D3	1E10	20%	7	Lin-D3	Cry-D3
	2C6	7%	7		
	1F4	−3%	7		
	2D7	−1%	7		
	6E5	0%	7		

GIA values are mean of 3 or more experiments against 3D7 strain.

^#^ 1F9 tested at 0.6 mg/ml in GIA.

Strain reactivity out of 7 allelic proteins by dot blot.

Binding location assigned by dot blot or western blot against linear and crystal domain chimeras.

[29] [30]. previously described AMA1 mAbs

QV-induced hybridoma supernatants were prescreened for cross-reactivity to the four vaccine homologous allelic proteins by ELISA and domain chimeras by dot blot (not shown). Representative mAbs against each domain, preferably those that cross-reacted with three or more allelic proteins, were expanded and tested in a GIA at 1 mg/ml against the 3D7 target strain. While some domain-1 mAbs were strain-specific and others cross-reactive, mAbs against domain-2 were exclusively strain-specific for 3D7 and mAbs against domain-3 were mostly cross-reactive (Table 1). The two previously characterized AMA1 mAbs 4G2 and 1F9 were accurately mapped by Western blot with chimeric proteins, to regions surrounding the hydrophobic trough (domain-2 loop and domain-1 respectively), validating the chimera based apporoach to epitope mapping. MAb 4G2 bound to chimera Lin-D2 and Cry-D1, and mAb 1F9 bound to Lin-D1 and Cry-D1 (Fig. 4A,B,C). Both of these mAbs were moderately inhibitory in a GIA against the 3D7 strain (Table 1). In contrast, three novel QV mAbs, 1B10, 4E8, 4E11, showed >60% inhibition and all three mapped to domain-1 on chimera Western blots. The domain-2 mAbs demonstrated low level inhibition (10% or less), while one of the domain-3 mAbs, 1E10, showed moderate inhibition, similar to mAb 4G2. The concentration of mAbs needed for 30% inhibition against the 3D7 target parasites (IC_30_ concentration) was about 10-fold lower for the three domain-1 mAbs, 1B10, 4E8 and 4E11 (0.15, 0.15 and 0.22 mg/ml, respectively) as compared to mAbs binding to other regions of AMA1 (mAb 4G2, 1.8 mg/ml; mAb 5A6, 3.5 mg/ml and mAb 1E10, 1.9 mg/ml) (Fig. S6). However, no individual mAb recapitulated the high level GIA activity of polyclonal anti-3D7 IgG in this assay (IC_30_ = 0.08 mg/ml).

### The most potent inhibitory mAbs mapped to the 1e-loop of AMA1 domain-1

Consistent with the published location of the mAb 4G2 epitope on the domain-2 loop, this mAb reacted with chimeras displaying the conserved face (CONS) and the domain-2 loop (D2+1e chimera) [31] (Fig. 4A,B,C). Likewise, mAb 1F9 reacted with chimeras displaying the C1L or 1d loop on the rim of the hydrophobic trough (HT) [29]. The novel domain-1 mAbs 1B10, 4E8 and 4E11 all had a similar reactivity pattern, mapping to the conserved face. These mAbs also reacted to the D2+1e chimera, displaying the *P. falciparum* domain-2 and 1e loops, but no reactivity to the Lin-D2 chimera containing the domain-2 loop was observed. This suggested that the epitope of the most potent domain-1 mAbs 1B10, 4E8 and 4E11 encompassed the 1e-loop (confirmed below). The moderately inhibitory domain-3 mAb 1E10 mapped to the polymorphic face (Fig. 4B,C).

### Broadly inhibitory AMA1 mAbs mapped to the conserved face and domain-3

Breadth of mAb recognition was tested by a dot blot against 7 AMA1 allelic proteins (Fig. 4D and Table 1). The domain-2 loop-binding mAb, 4G2, and the novel domain-3 mAb, 1E10, bound to all 7 AMA1 alleles. In a parallel invasion assay these two mAbs weakly inhibited the corresponding parasite strains, confirming that they recognized strain-conserved, broadly inhibitory epitopes (Fig. 4E). The three most potent 1e-loop mAbs (1B10, 4E8 and 4E11) recognised most but not all protein variants. GIA confirmed these results as strain W2mef escaped inhibition by mAbs 1B10 and 4E11, and strain M24 was refractory to inhibition by all three 1e-loop mAbs. A negative control mAb, 58F8 which recognizes the N-terminal region of AMA1, did not show significant invasion inhibition and mAb 5A6, which bound to a strain-specific domain-2 epitope, inhibited only the 3D7 strain (Fig. 4E).

Since mAbs 1B10, 4E8 or 4E11 bound to 3D7 AMA1 but not to M24 AMA1, we analyzed the polymorphic differences between 3D7 and M24 AMA1 and found a single difference at residue 230 within the 1e-loop. When residue 230 of the 3D7 AMA1 ectodomain displayed on phage was mutated to alanine all three mAbs showed reduced reactivity to the mutant phage compared to phage displaying wild-type 3D7 AMA1 (Fig. S7). This confirmed that the 1e-loop region was the target of the most potent AMA1 mAbs (1B10, 4E8 and 4E11).

### AMA1 antibodies targeted two different biological processes

**Figure 5:**
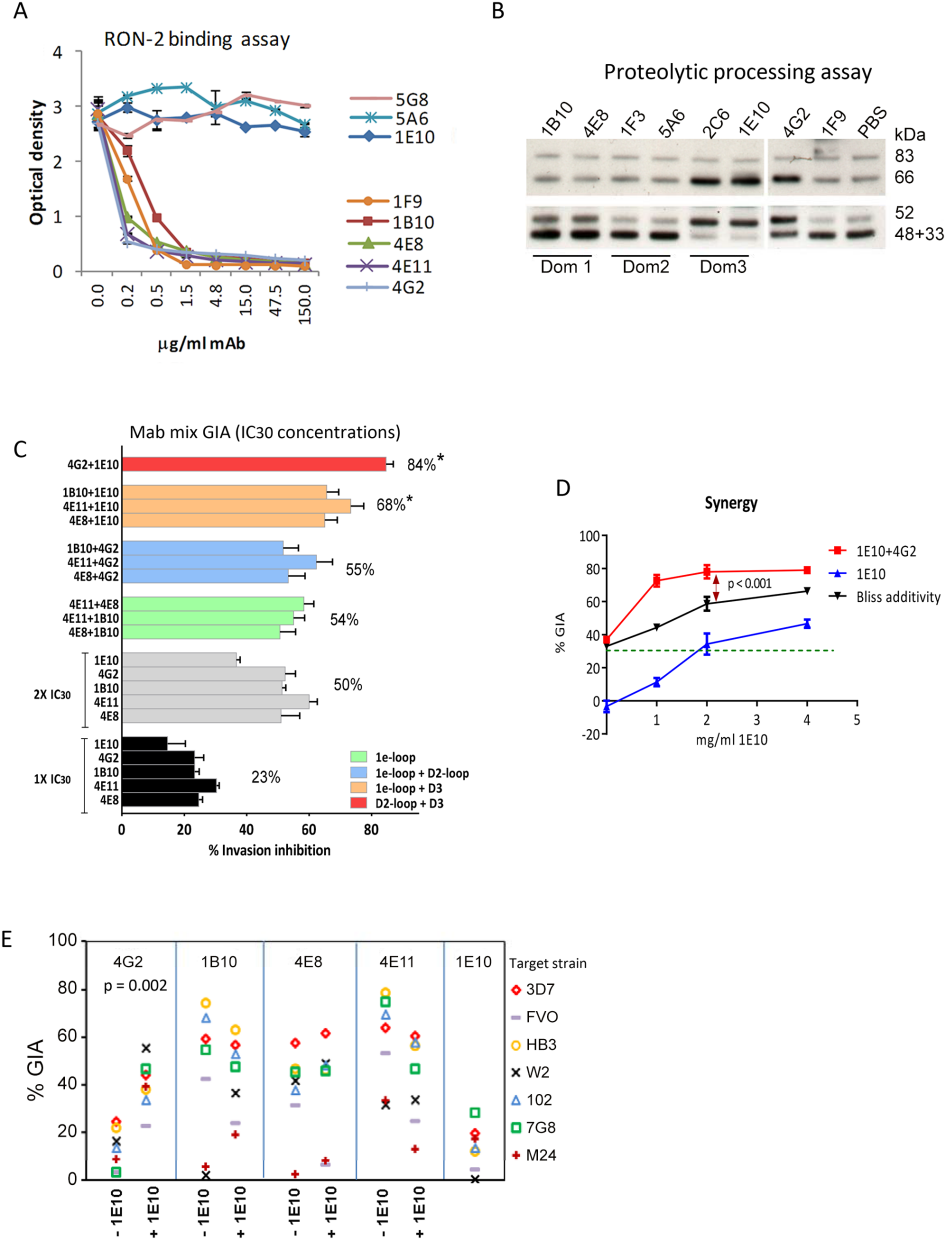
Biological activity of monoclonal antibodies. (**A**) Binding of 3D7 AMA1 (OD_450_) to immobilized RON2 peptide inhibited by serial dilutions of the mAbs. Negative control mAb 5G8 binds to the N-terminal prosequence. (**B**) Western blot of a 3D7 parasite processing inhibition assay using 200 µg/ml mAbs. Top panel shows the merozoite bound full-length (83 kDa) 3D7 parasite AMA1 and the product of N-terminal processing (66 kDa). Bottom panel shows the co-migrating products of normal shedding (48+44 kDa) and the product of anomalous AMA1 processing (52 kDa). These fragments were captured from the culture supernatant using a sub-inhibitory concentration of polyclonal anti-3D7 AMA1 sera (1∶2500) in the processing assay [34]. (**C**) GIA against 3D7 target strain, using 1×IC_30_ dose of individual mAbs (black), 2×IC_30_ dose of individual mAbs (gray), 1×IC_30_+1×IC_30_ mixture of two 1e-loop mAbs (green) or 1e-loop+domain2-loop mAb (blue) or 1e-loop+domain-3 mAb (orange) or domain2 loop+domain-3 mAb (red). Mean+s.e.m. of 3 experiments; (*) p<0.05 comparing the mean of each group to the mean of 2×IC_30_ dose of individual mAbs (gray bars). (**D**) GIA against the 3D7 parasite strain using increasing concentrations of mAb 1E10, with (red line) or without (blue line) the addition of 1×IC_30_ concentration of mAb 4G2 (1.8 mg/ml, expected 30% GIA in green). Predicted inhibition for additive interaction (black line) was calculated according to “Bliss independence” as has been applied to determine synergy by Williams *et al.* [55] [56]; data are mean+s.e.m. of triplicate wells. (**E**) Inhibition of 7 parasite strains using 2 mg/ml of the RON2 inhibitory mAb or a mixture of 1 mg/ml each of the RON2 inhibitory mAbs and processing inhibitory mAb 1E10; a representative of two experiments is shown.

GIA activity of AMA1 antibodies has been associated with inhibition of two biological processes: RON2 protein binding and AMA1 proteolytic processing. Representative mAbs against all three domains were analyzed to determine if they blocked the interaction of AMA1 with its receptor, RON2 [24], [25], [52], or if they could inhibit the proteolytic cleavage of the 66 kDa membrane bound AMA1 to the 48+44 kDa soluble forms which are shed [22], [34] [53]. The mAbs that bound to loops adjacent to the hydrophobic trough (1F9, 1B10, 4E8, 4E11, 4G2) blocked the binding of RON2 peptide to AMA1 (Fig. 5A). RON2 binding was not altered by mAbs that bound to domain-2 (mAb 5A6), domain-3 (mAb 1E10), or the N-terminal pro-domain (mAb 5G8). Secondary proteolytic processing of AMA1 on 3D7 strain parasites was blocked by mAbs binding to domain-3 (2C6, 1E10). Inhibition of processing was indicated by increased intensity of the merozoite surface associated 66 kDa form combined with reduced intensity of the products of normal processing (co-migrating 44+48 kDa bands). Inhibition of normal processing also resulted in increased levels of the 52 kDa product of anomalous AMA1 processing [54] [34]. In contrast, mAbs binding to domain-1 (1B10, 4E8, 1F9), or domain-2 (1F3, 5A6) did not inhibit AMA1 processing (Fig. 5B). Some alteration of processing was also detectable in presence of the mAb 4G2, probably due to the proximity of the base of the domain-2 loop to the C-terminal processing/shedding site at Thr_517_ (Fig. 4B) [23].

### A domain-3 antibody enhanced the inhibitory activity of broadly inhibitory conserved face antibodies

To test if broadly inhibitory antibodies showed additivity or synergistic inhibitory effects, we analyzed selected mAbs in a GIA against 3D7 parasites at their respective 1×IC_30_ concentration (black bars; average inhibition, 23%) and at 2×IC_30_ concentration (gray bars; average inhibition, 50%) (Fig. 5C). When pairs of mAbs binding to spatially proximal epitopes were mixed at their respective 1×IC_30_ concentrations (1e-loop mAb mixtures in green or 1e-loop+domain-2 loop mAb mixtures in blue), the resulting inhibitions were not different from the 2×IC_30_ concentration of individual mAbs. However, when mAbs binding to spatially distant epitopes were mixed at their IC_30_ concentration (1e-loop+domain-3 mAbs in orange or domain-2 loop+domain-3 mAb in red), the average inhibitions were significantly higher than that of the 2×IC_30_ concentration of individual mAbs (p<0.05 corrected for multiple comparisons). The most potent inhibitory combination, mAb 1E10+4G2, was tested to confirm synergy using the “Bliss independence” equation recently used to discern synergistic antibody combinations by Williams *et al.* [55], [56]. In a GIA against 3D7 parasites, a fixed IC_30_ concentration of mAb 4G2 was mixed with a range of concentrations of mAb 1E10 (Fig. 5D) and synergy was assumed if the combination inhibited better than predicted by Bliss independence. The observed inhibition of the 4G2+1E10 mAb combination (red line) was higher than the predicted GIA activity (black line), thus confirming synergy (ANOVA followed by Tukeys's test, p<0.0001). In a GIA against 7 diverse parasite strains, only the mAb 1E10+4G2 combination showed enhanced inhibition across strains (Fig. 5E, t test, p = 0.002). Thus domain-3 antibodies, which by themselves were not potent inhibitors, could synergize with antibodies binding to a strain-transcending epitope on the conserved face, domain-2 loop.

### QV enhanced the immune response against domain-3 and the conserved face epitopes

**Figure 6:**
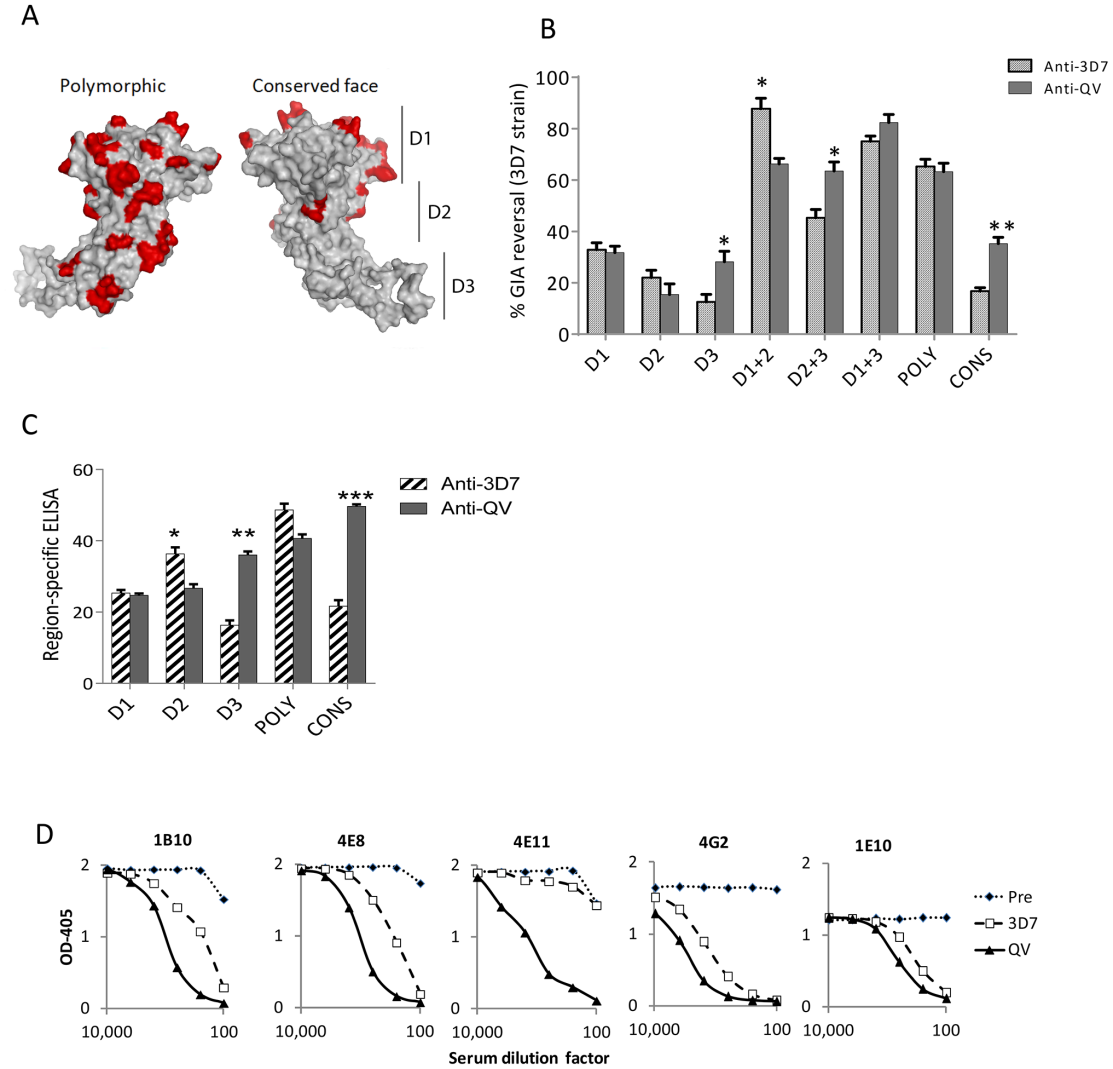
Mapping inhibitory activity of polyclonal antibodies. (**A**) Distribution of high frequency polymorphisms on the three domains (D1, 2, 3), the polymorphic face and the conserved face of AMA1. (**B**) Region-specific inhibitory contributions determined by adding chimeras to reverse anti-3D7 or anti-QV serum pool mediated GIA activity (approximately 60% starting GIA activity) against 3D7 parasites. Reversal using chimeric proteins CryD1, CryD2, CryD3, Cry D1+CryD2, CryD2+CryD3, CryD1+CryD3, POLY and CONS (4 µM, ∼200 µg/ml final concentration) was determined with respect to *P. berghei* AMA1 as the control. Mean+s.e.m. of 3 experiments and (*) indicates statistical significant p value of t-tests comparing anti-3D7 and anti-QV reversals. (**C**) Region-specific ELISA response with pooled sera (% of total) calculated as the ratios of end-point titers against a 3D7 chimera relative to the end-point titer against 3D7 AMA1 protein (mean+s.e.m. of triplicates in a representative of three experiments). (**D**) Competition ELISA shows the binding of HRP labeled mAbs to heterologous 102-1 AMA1 protein. The mAb binding was competed out using serial dilutions of polyclonal anti-3D7 or anti-QV or pre-immune rabbit serum pools (*x axis*). Shown is mean OD_405_ of 2 wells from a representative of two experiments.

Using the strain-specific anti-3D7 as the reference, we conducted differential mapping of the polyclonal anti-QV inhibitory response. In a GIA against 3D7 strain, equivalent final concentration of 3D7 chimeric proteins CryD1, CryD2, CryD3, CryD1+CryD2, Cry D2+CryD3, CryD1+CryD3, CONS and POLY were added to deplete region-specific antibodies against domains-1, 2, 3, 1+2, 2+3, 1+3, conserved face and polymorphic face, respectively (Fig. 6A). The extent of GIA reversal was used to dissect region-specific inhibitory contributions (Fig. 6B). For the anti-3D7 IgG, mAb mapping data would have predicted domain-1 to have the highest inhibitory contribution, however, the D1 chimera caused only 33% reversal as compared to 87% reversal by the mixture of D1 and D2 chimeras. This result was not surprising because vaccination with AMA1 domains has previously shown that antibodies to these two domains are needed for high level GIA [57]. Between the two faces of AMA1, the polymorphic face contributed more towards the inhibition (65% reversal) than the conserved face antibodies (16% reversal).

Comparing anti-3D7 and anti-QV GIA reversal showed increased levels of cross-reactive antibodies in anti-QV correlated with increased GIA reversal by chimera combinations that contained domain-3 (t test; D3, p = 0.0095; D2+3, p = 0.0092) and the overall reversal for D1+3 chimera was the highest for anti-QV (Fig. 6B). Conversely, D2 (not statistically significant) and D1+D2 (p = 0.0035) responses for anti-QV were lower than anti-3D7. Between the two faces, the response to polymorphic face was unchanged while enhanced conserved face inhibitory contribution was observed in anti-QV (p = 0.0006). A region-specific ELISA using chimeric proteins as coat antigens also showed that QV induced higher levels of domain-3 (p = 0.0002) and conserved face (p<0.0001) antibodies and reduced domain-2 antibodies (p = 0.008) (Fig. 6C) and another region-specific ELISA using the M24 AMA1 affinity purified anti-3D7 and anti-QV IgG (tested in Fig. 2C), confirmed this observation (Fig. S8). Thus, as compared to the strain-specific monovalent 3D7 AMA1 vaccine, vaccination with QV enhanced the immunogenicity of two less-polymorphic regions of AMA1: the conserved face and domain-3 while the response to domain-2 was reduced. Since the inhibitory activity of the polymorphic face was unchanged between anti-3D7 and anti-QV, it can be concluded that QV still generated a component of partially strain-specific antibodies targeting polymorphic areas on each strain and additionally it elicited higher levels of cross-strain inhibitory antibodies that mapped to domain-3 and conserved face.

A mAb competition ELISA was performed to determine the ability of anti-3D7 and anti-QV serum pool to inhibit the binding of labelled cross-reactive mAbs (1B10, 4E8, 4E11, 4G2 and 1E10) to a non-vaccine strain 102-1 AMA1 (Fig. 6D). A lower concentration of anti-QV was required to compete out mAbs 1B10, 4E8, 4G2, 1E10 and, strikingly, antibodies competing for at least one broadly inhibitory epitope defined by mAb 4E11 epitope on the conserved face 1e-loop were present only in anti-QV, providing further proof of a partial shift of immunogenicity in favor of conserved epitopes.

## Discussion

Given the failure of AMA1 vaccines to protect broadly, this study investigates the molecular basis of anti-AMA1 mediated cross-strain invasion inhibition. Using a mixture of antibodies against monovalent vaccines, Drew *et al.* have suggested that overlapping strain-specific antibodies can achieve broad inhibition [43]. We show that vaccinating with a mixture of four divergent AMA1 allelic proteins (3D7, FVO, HB3, and W2mef) can outperform the GIA activity of a mixture of monovalent antisera (Fig. 1F) and whilst overlapping strain specific antibodies almost certainly contributed, QV vaccination achieved a kind of mixed-allele immunogenicity bonus, similar to that reported by mixing three alleles of the Duffy binding protein vaccine [58]. The enhanced cross-strain inhibition by anti-QV was associated with no change in polymorphic face response, decrease in domain-2 response and increased response to two relatively non-polymorphic regions, the domain-3 and the conserved face. Reduction in domain-2 response was interesting because polymorphisms within domain-2 are important for antigenic escape *in vitro* [28], [37] and molecular analysis of a Phase 2b trial showed active selection at a domain-2 polymorphic site [40]. Not surprisingly, all three domain-2 mAbs identified here were also strain-specific (Table 1). Our findings support the further development of multivalent AMA1 vaccines because broad antigenic coverage is achieved not only by expanded antigenic footprint of strain-specific antibodies but by inducing higher levels of antibodies against conserved inhibitory epitopes [41], [42].

While we know of no mechanisms or precedents that would definitively explain our finding that co-administering polymorphic allelic AMA1 proteins increases the immunogenicity of subdominant conserved epitopes, a simple hypothesis based on the observations in influenza can be advanced. During serial influenza virus infections, memory B cells specific for the more abundant strain-specific epitopes on the head of hemagglutinin (HA) are readily expanded while fewer B cells specific for the conserved stem epitopes are crowded out. When a pandemic strain infects that contains a very different HA head, the strain-specific epitopes are replaced by new epitopes but exposure to the conserved stem epitopes continue [59]. Thus serial exposure to diverse viruses enhance antibody responses to conserved stalk epitopes of hemagglutinin [60]–[62], even though the polymorphic HA head epitopes are typically more dominant. Likewise, in AMA1 the more abundant strain-specific epitopes, which may also have a higher affinity for T or B cell receptors, compete better for space on antigen presenting cells, but when strain-specific epitopes are diluted out by mixing of diverse AMA1 proteins, the undiluted concentrations of conserved epitopes can crowd out the polymorphic responses to attract and activate more T and B cells. Indeed, strain-broadening [63] and increased domain-3 responses [64] have been reported in the higher age groups among malaria exposed populations and it may be possible that QV vaccination in children could confer immuno-protection similar to that achieved in adults.

Defining broadly inhibitory epitopes will be critical to apply structural vaccinology approaches to improve the breadth and GIA activity of all AMA1 vaccines. The 1e-loop region (residue 225–235) was identified as the most susceptible mAb target on AMA1. The four QV alleles display the most prevalent polymorphism combination within the 1e-loop (N_228_, K_230_; ∼70% of alleles). It is currently unclear why 1e-loop was the most susceptible target. As compared to the relatively flexible 1b, 1f and domain-2 loop, the loops at the other end of the hydrophobic trough, 1c and 1d/C1L and 1e, are less flexible [29]. In particular the 1e-loop is bounded on one side by the hydrophobic trough, and on another side by a pocket that forms a critical contact with Arg_2041_ of RON2 [25], [65]. We speculate that the reduced flexibility of the 1e-loop and its proximity to residues involved in RON2 binding are the basis of higher inhibitory activities. Moreover, the intimate nature of the interaction of RON2 wrapping around the 1e-loop places a functional constraint that limits the selection for polymorphisms in recessed regions, but does allow for polymorphisms at the apex of the loop.

In addition to the most potent antibodies mapping close to the receptor binding region, a domain-3 mAb 1E10 was found to be moderately inhibitory yet strain-transcending. Similarly, mAb DV5 generated against a loop peptide on the polymorphic face of domain-3 was shown to be inhibitory [66]. The synergy between mAbs 1E10 and 4G2 was consistent with a report demonstrating that mutations preventing AMA1 processing render the parasites more sensitive to inhibition by RON2-targeting antibodies [67] and this may be the first example of synergy between two mAbs targeting two functional regions on the same malaria antigen. Most importantly, this synergistic mAb combination bound to regions whose immunogenicity was boosted by immunization with the QV. A similar enhancement in neutralization coverage was reported using a combination of HIV mAbs targeting the CD4 binding site and the variable loops-1 and 2 of the *Env* protein [68].

While the most potent inhibitory QV mAbs were directed against epitopes on domain-1, the chimera mapping of polyclonal sera indicated that inhibitory activity of anti-QV was not concentrated on domain-1 alone. This apparent contradiction may result from differences in mouse and rabbit responses [69], but we also expect polymorphic face epitope gaps in our mAb library due to the screening in favor of cross reactive mAbs. The higher overall GIA activity of the polyclonal antibodies (Fig. S6) as compared to mAbs, suggests the possibility of a complex interplay of antibodies to multiple domains [57], [70], that were not yet fully investigated using mAb combinations. Despite the differences there were several similarities between the two sets of mapping data. Chimera mapping of anti-QV, showed domain-2 to be the least important for cross-strain inhibition; likewise, no cross-reactive inhibitory domain-2 mAbs were identified. Among the paired combinations, the most potent reversal was caused by depleting the domain-1+3 antibodies from anti-QV, again consistent with the observed inhibitory effects of domain-1 and synergistic domain-3 mAbs.

Attempts to engineer an immunogen to elicit high levels of cross-reactive antibodies by silencing a dominant strain-specific epitope on the C1L loop led to loss of immunogen potency [71]. The C1L loop genotype is associated with *in vitro* susceptibility in GIA [28], [37], [43], susceptibility to malaria in the field [18], and escape from AMA1 vaccine effect in humans [15], [40]. Therefore an ideal AMA1 vaccine would induce high levels of broadly inhibitory antibodies and strain-specific antibodies to match the dominant serotypes. In our studies, anti-QV titers, IC_50_ and overall GIA activity were no worse than that induced by monovalent vaccines against homologous strains. In contrast, a mixture of two artificially designed DiCo proteins showed lower reactivity against naturally occurring allelic proteins [70], and anti-DiCo mix GIA activity was lower than that induced by natural allele mix antibodies, tested against homologous strains [42]. While both natural allelic proteins and DiCo mix enhance broadly inhibitory antibodies, combinations of polymorphisms within naturally occurring allelic proteins provide an extra measure of inhibitory coverage against their respective homologous strains. Thus far, this matching of polymorphic combinations between the vaccine and the target strain has been shown to be associated with high level GIA, protection in animals [8], [13] and in humans [15]. Hence our results support the use of natural allelic proteins to overcome AMA1 diversity.

The most significant improvement in GIA breadth is observed when going from a bivalent to a trivalent vaccine [19], [41]. We found a smaller but still significant increase in vaccine breadth on going from trivalent to QV. Kusi *et al.* have reported little improvement going from a 3 protein DiCo mix to a 7 DiCo/natural allele mix [72]. Hence, there appears to be a diminishing marginal improvement in antigenic breadth above 3 proteins and polymorphism dilution beyond an optimum may render the polymorphic residues too dilute to induce functional type-specific antibodies. It is unclear what that optimum number might be. Miura *et al.* showed a 5-allele vaccine was better than a 4-allele mix, and adding a sixth allele made no further improvement [37]. It is notable, that the three vaccine groups received different antigen doses and this could complicate the interpretation of their results. As compared to the homologous inhibition against 3D7 target, the strains MT/S1, C2A, GB4 and DD2 (homologous to W2mef) were less susceptible to the 5-allele vaccine antibodies, reported by Miura *et al.* [37]. In our study these strains were inhibited similar to the vaccine homologous strain HB3 by anti-QV in a GIA conducted by the NIH reference laboratory, at equivalent total IgG concentration (Fig. S4A). Anti-QV also potently inhibited all five *P. falciparum* isolates obtained recently from Cambodian malaria patients [55]. While these results are encouraging, numerical comparisons of GIA results are likely to be confounded by differences in animals, adjuvants, antigen dose, delivery platform and GIA methodology. Only head to head trials can definitively establish how the breadth and potency of QV compares to other AMA1 vaccines or other blood stage vaccine candidates such as Rh5, which induces broad inhibition using a single allelic component [73]. Limiting resources and dwindling field sites available to test blood stage vaccines may soon necessitate such comparisons. Nevertheless, rabbit anti-QV was pan-reactive by ELISA and it inhibited 22 non-vaccine parasite strains that included recent field isolates. Our data taken together with the findings of Drew *et al.* [43] make a strong case for further development of QV.

Also of significant note, the level of antibody response and GIA activity induced in rabbits, has not yet been achieved in humans, either using recombinant proteins [74], [75] or viral vectored antigens [76] [77]. We anticipate that the identification of inhibition susceptible epitopes will provide a rational framework to design immunogens that induce antibodies with broad reactivity and low IC_50_, as this may further reduce the number of components required. Another cause of concern has been that polyvalent vaccines, particularly those against viral pathogens, often require geographic and periodic adjustment to match the circulating strains [78]. Although it remains to be seen if a future AMA1 vaccine would also require periodic reformulation, several differences between malaria parasites and viruses suggest that this may not be necessary. AMA1 populations from distant endemic areas have similar diversity [35], [79] and, although the *in vitro* and *in vivo* immune pressure can rapidly select for viral escape mutants [80], [81], mutations in the AMA1 gene have not been detected during prolonged culture or among parasite lines serially maintained in AMA1 immunized mice [82]. It is assuring that a major mechanism of broadened inhibition involves refocusing antibody responses towards regions that are structurally conserved. Overall, we agree with previous reports that the vast diversity of AMA1 may be overcome by a vaccine that contains only a few allelic components [37], [38], [43]. In future, human-use formulations of QV need to be evaluated in primate models and in a human blood-stage challenge model [83]. If successful, these trials could lead to QV being combined with the partially efficacious pre-erythrocytic stage malaria vaccine candidate RTS,S [84].

## Materials and Methods

### Ethics statement

Rabbit immunizations were performed at Spring Valley Laboratories (SVL) (USDA License No. 51-R-0051). SVL animal research facility is accredited by the Association for Assessment and Accreditation of Laboratory Animal Care International (AAALACi). The vaccine protocol (SVP10-0059) was approved by the SVL Animal Care and Use Committee. The views mentioned are that of the authors and not of the U.S. Department of the Army or Department of Defense.

### Diversity analysis

Full-length AMA1 sequence of 175 field isolates (Table S1) and 26 culture adapted strains were aligned by CLUSTAL (Lasergene software). AMA1 diversity was visualized on a dendrogram created using Dendroscope software (http://ab.inf.uni-tuebingen.de/software/dendroscope/).

### Expression and purification of recombinant AMA1 proteins

Genes encoding 449 amino acid *P. falciparum* FVO, M24, 7G8 and 102-1 AMA1 ectodomain (residues 83–531) were codon optimized and the proteins were expressed in *E. coli* essentially as described previously [17]. The production of HB3 and W2mef AMA1 proteins (residues 25–546) has been described previously [9], [14].

### *P. falciparum-P.berghei* AMA1 chimeras

Crystal structures of AMA1 (PDB references 1W81, 1Z40, 2Q8A) were used to design continuous surface chimeric proteins that displayed the various three dimensional structural elements of *P. falciparum* AMA1 on the *P. berghei* AMA1 scaffold. Chimeras were based on AMA1 residues 83_Gly_ to 531_Glu_ of *P. falciparum* 3D7 sequence (accession number XP_001348015) and *P. berghei* AMA1 ANKA strain sequence (XP_678057 or CAH96497). The chimeras displaying the domain1, domain2, domain3, the hydrophobic trough, the polymorphic face, conserved face and the domain2+1e loop were termed as CryD1, CryD2, CryD3, HT, POLY, CONS and D2+1e respectively [32], [33]. To avoid potential steric clashes CryD1, CryD2 and CryD3 were designed with overlapping (∼7 Å) *P. falciparum* regions (Table S2). Chimeric proteins displaying the linear domains of *Plasmodium falciparum* AMA1 on the *P. berghei* AMA1 scaffold were also produced. To make the linear domain chimeras, sequences of *P. falciparum* AMA1 gene were PCR amplified using a synthetic gene template of *P. falciparum* AMA1 (residues 83_Gly_ to 531_Glu_, accession number AAB36701). Scaffold sequences were amplified from the native *P. berghei* AMA1 genomic DNA using primers that overlapped the *P. falciparum* DNA fragments. Full-length chimeric genes were then assembled by a PCR stitch reaction using external primers. Residue boundaries 83_Gly_–303_Cys_ were treated as domain-1, residues 304_Arg_–418_Cys_ as domain-2 and residues 419_Leu_–531_Glu_ as domain-3. Five linear domain chimeras displaying the three domains: LinD1, LinD2, LinD3, LinD1+2 & LinD2+3 were produced. The genes for the chimeric proteins were cloned in pET32 based plasmid, expressed in *E. coli* Tuner strain, grown in superbroth and protein expression was induced by 0.5 mM IPTG for 2 hrs. The chimeric proteins were purified using Ni-NTA affinity, refolded and the purified over an anion exchange column. Mixtures of these chimeric proteins were tested in GIA reversal assays (for example CryD1+CryD2+CryD3 or POLY+CONS) to ensure that together these combinations covered all of the inhibitory epitopes of 3D7 AMA1protein, as was evidenced by ∼100% GIA reversal (Fig. S5C). *P. berghei* AMA1 showed negligible reversal.

### Rabbit immunization

Three rabbits per group each received three doses of 100 µg AMA1 vaccine per dose emulsified in Montanide ISA720 (Seppic Inc, Paris). The Quadrivalent vaccine (Quadvax) consisted of 25 µg each 3D7, FVO, HB3 and W2mef proteins; trivalent vaccines contained 33 µg of three allelic proteins and bi-allelic vaccine contained 50 µg of two allelic proteins. Emulsification was achieved by vigorous vortexing for 10–15 min and 1 ml vaccine was administered at multiple sites, subcutaneously, on the animal's back at four week intervals. Rabbits were bled out 2 weeks after the third vaccination. Sera were heat inactivated and stored at −70°C until used for invasion inhibition assays.

### Affinity purification of IgG

Pooled sera (1.33 ml from each of the 3 rabbits within a group) was diluted 5-fold in IgG binding buffer (Pierce, Rockford, IL), and passed over a 1 ml Protein G column (GE, Pittsburgh, PA), the column was washed and bound IgG was eluted using IgG elution buffer (Pierce), neutralized with 1M Tris HCL pH 8.0, dialyzed against PBS and quantified by measuring OD_280_. Five mg of 3D7 or M24 AMA1 protein was covalently linked to 1 ml cyanogen bromide sepharose column according to the manufacturer's instructions (GE). Anti-AMA1 IgG was bound and eluted from this affinity column as described above.

### ELISA

ELISA protocol has been previously described [17]. End-point titer was the dilution that gave OD_405_ = 0.5. Region-specific antibody titer was defined as: (End-point titer against a domain chimera/end-point titer against the full-length 3D7 AMA1 protein)×100.

### 1-cycle Flow-cytometric GIA (WRAIR method)

All inhibition assays in duplicate wells were performed by this method, unless stated otherwise [44]. Synchronized cultures at late-ring stage were diluted to 0.25–0.3% parasitemia and 2% hematocrit by using uninfected cells. The sera were heat inactivated before use and final culture volume was 60 µl. Parasites developed for 40 h at 37°C in static cultures and ring stages formed after the invasion cycle were stained with 1×SYBR green dye (BMA, Rockland, ME) and counted by using BD FACSCalibur flow-cytometer. Controls wells were matched for the test strain and contained equivalent volume of adjuvant immunized serum control or PBS (for mAb and IgG GIA). Percent inhibition of invasion = 1−(% parasitemia in test well/% parasitemia in control well).

### GIA reversal using allelic proteins

Serum pool of anti-QV or anti-3D7 AMA1 were diluted to give ∼60% inhibition of the 3D7 parasite. AMA1 allelic proteins derived from 3D7, FVO, HB3, W2mef, 102-1, 7G8, or M24 strains (∼150 µg/ml or 2.8 µM) were added to selectively deplete cross-reactive antibodies. Immunologically non-reactive *P. berghei* AMA1 protein showed no inhibition reversal, while the homologous 3D7 AMA1 showed complete reversal. GIA reversal = (inhibition in presence of 2.8 µM *P. berghei* AMA1−inhibition in the presence of 2.8 µM test protein)/inhibition in presence of *P. berghei* AMA1 at 2.8 µM.

### GIA reversal using the chimeras

Region-specific inhibitory contribution of antibodies was determined using protein chimeras to reverse anti-QV or anti-3D7 AMA1 mediated inhibition of 3D7 parasites. To ∼60% inhibitory dose of serum pool, 4 µM chimeric proteins (∼200 µg/ml) were added. Percent GIA reversal = (inhibition in presence of 4 µM *P. berghei* AMA1−inhibition in the presence of 4 µM test chimera)/inhibition in presence of 4 µM *P. berghei* AMA1.

### 2-cycle, purified IgG invasion inhibition assay (Burnet Institute method)

*P. falciparum* growth inhibition assay was performed as described previously [46], [85]. Parasites were allowed to develop through two cycles of erythrocyte invasion for 72 hours at 37°C, stained with SYBR green dye (Invitrogen) and infected cells counted using a FACSCantoII Flow-cytometer (BD). FACS counts were analyzed using FloJo software (Ver 6.4.7). Percent inhibition of invasion = 1−(% parasitemia in test well/% parasitemia in medium control well). All GIAs were run in a 96-well plate format, with each antibody tested in duplicate wells. Parasite growth inhibition is represented as the combined mean of two separate duplicate well assays set up on different days.

### 1-cycle, purified IgG invasion inhibition assay (NIH reference center method)

IgGs from rabbits were purified from pooled sera using protein G columns (Pierce Inc., Rockford, IL); the eluted fractions were dialyzed against RPMI 1640 (Life Technologies, Gaithersburg, MD) and concentrated with centrifugal filter devices (Millipore, Billerica, MA). The purified IgGs were preadsorbed with uninfected human O+ erythrocytes, sterilized by filtration through a 0.22-µm filter and heat inactivated at 56°C for 20 min before use in the assay. Late trophozoite and schizont stages of *P. falciparum* were allowed to develop and invade in the presence of either test or medium only control [45]. Cultures were maintained for 40 to 42 h and relative parasitemia was determined by biochemical determination of parasite lactate dehydrogenase. Percent inhibition of the immune IgG was calculated as 100−[(A_650_ of test IgG−A_650_ of normal RBCs)/(A_650_ of infected RBCs without any IgG−A_650_ of normal RBCs)×100].

### Monoclonal antibodies (mAb)

Monoclonal antibodies were developed by immunizing 3 mice multiple times with QV using the Precision Antibody's immunization technology (Columbia, MD). Target specific antibody titers were determined by ELISA and a fusion was performed with B-cells from splenocytes and lymphocytes. The myeloma partner was derived from the cell line P3X63Ag8.653. Fused cells were selected in a HAT media and grown from a single cell. Hybridoma clone supernatants were screened by ELISA for reactivity to the four allelic proteins 3D7, FVO, HB3 and W2mef AMA1. Out of the total 38 clones obtained representative mAbs against all three domains were picked, preferably if they reacted to multiple allelic proteins. Selected mAbs were expanded *in vivo* using athymic nude mice and mAbs were purified from the ascetic fluid using a Protein G column (GE Healthcare). Other mAbs used in the study were: rat mAb 4G2dc1 that recognizes a cross-reactive conformational epitope [30]; rat mAb 58F8dc1 that recognizes the N-terminal region present only on unprocessed AMA1; and mouse mAb 1F9 which binds to the residues on the C1L loop of 3D7AMA1 [12]. Mab 4G2 and 58F8 were gifts from Dr. Clemens Kocken, Biomedical Primate Research Center, Rijswijk, The Netherlands.

### Immuno-blot

1 µg of the AMA1 proteins under non-reducing conditions was electrophoretically transferred to a nitrocellulose membrane immune-blots were performed essentially as described previously [17].

### RON2 peptide competition ELISA

Two µg/ml of RON2 peptide labeled with biotin at the N-terminus was immobilized on streptavidin plates (Thermal Fisher), followed incubation in BLOTTO Blocking Buffer (Pierce, Rockford, IL) for 1 hr. An equal volume of 0.0015 µg/ml of 3D7 AMA1 and decreasing concentrations (150 µg/ml to 0.15 µg/ml) of mAbs (1E10, 1B10, 4E8, 4E11, 5A6, 1F9, 4G2 and 5G8) were added to the well. After 1 hr incubation the wells were washed and 1∶5000 dilution of rabbit anti-AMA1 polyclonal serum was used to detect bound AMA1. ABTS substrate was added to the well after 1 hr incubation OD_450_ was recorded.

### Mutagenesis of Lys_230_ to Ala in the 3D7 form of AMA1

Mutagenesis of Lys_230_ to Ala was carried out by the technique of splice overlap extension. PCR was used to amplify overlapped DNA fragments from the 3D7 AMA1 ectodomain template in PHENH6 plasmid such that both PCR fragments contained the K_230_ mutation. The splice overlapped PCR was performed using PHENH6 forward and reverse primers that incorporate the flanking region from PHENH6. Preparation of phage clones and phage ELISA against the mAbs was essentially as described previously [12].

### AMA1 processing inhibition assay

The processing inhibition assay on 3D7 strain parasites was performed essentially as described previously at 200 µg/ml final mAb concentration [86]. Merozoite pellets were harvested and analyzed for membrane-associated forms of AMA1, while soluble forms were trapped by including a non-inhibitory concentration of anti-3D7 AMA1 rabbit serum (1∶2500 dilution) in the processing assay. Proteins were run on a non-reducing SDS-PAGE and AMA-1-specific bands were stained as described [34].

### Monoclonal Competition ELISA

MAbs were labeled using Lightning-Link® Horseradish Peroxidase kit (Innova Biosciences, Cambridge UK). AMA1 protein of 102-1 strain was coated on ELISA plates (100 ng/well). Wells were blocked with 1% casein blocker for 2 hrs, washed with PBS-Tween and then 50 µl individual rabbit serum dilutions were added to the wells for 1 hr. To the same well, 50 µl of HRP-labeled mAbs, diluted to yield 1–1.5 OD_405_, were added and incubated for 1 hr. Plates were washed and ABTS substrate was added. After 1 hr incubation, stop solution was added and plates were allowed to sit for 5 min before the OD_405_ was recorded.

### Statistical analysis

To compare differences in GIA responses against different strains the data from individual rabbits in each of the vaccine group (explanatory variable) from 3 independent experiments (repeat component) was analyzed by Repeat Measures Multivariate Analysis of Variance (MANOVA). For ELISA the individual rabbit data was analyzed by MANOVA to discern differences between groups. Dunnett's method is used to adjust p-values for the *post hoc* testing, when comparing all groups to the QV group. Analysis of Variance (ANOVA) was used if the rabbit data were pooled and p values adjusted using either Dunnett's method (if all groups were compared to the QV) or Tukey's method (for all pair-wise comparisons). When two groups of data were to be compared a 2-sample t-test was used. ELISA data was log_10_ transformed to obtain close to normal distribution before statistical analysis. Group analysis was conducted using SAS software version 9.3. Correlation between sequence distance and GIA was analyzed by linear regression. For synergy analysis, GIA over a range of 1E10 concentrations (0–4 mg/ml) was measured against 3D7 parasites in the presence or absence of an IC_30_ concentration of mAb 4G2 (1.8 mg/ml). The observed inhibition by the mixture was compared to that predicted by an equation for Bliss independence as was applied to GIA by Williams *et al.* [55], [56]. GIA_additive_ = [1−(1−% GIA_1E10_)*(1−%GIA_4G2_ at its IC_30_)]. GIA dose response curves were used to predict the concentration of antibody that would give either 50% or 30% inhibition using non-linear curve function within Graphpad Prism^R^ software.

## Supporting Information

Figure S1 **Inverse relationship between sequence distance and GIA.** Mean invasion inhibition data from 3 rabbits was plotted against the amino acid difference between the vaccine and each of the 8 target strains (sequence distance), tested by the WRAIR GIA method. For anti-QV, the amino acid difference of a heterologous strain was against the most similar QV allele. Lines of best fit (solid black), error range (dotted black and pink), P value indicating if the slope is significantly non-zero, R^2^ and 95% CI of the slope (m) are shown.
(TIF)

Figure S2 **GIA with affinity purified antibodies used to calculate the IC_50_.** Anti-3D7 and anti-Quadvax IgG were affinity purified over a 3D7 AMA1 column. Bound/eluted (bound; red and blue lines) or the flow-through fractions (FT; orange and green lines) were adjusted to equivalent IgG concentration and tested against 3D7, FVO and M24 parasite strains. Mean+s.e.m. of 3 independent experiments against 3D7 and FVO strains and one experiment in triplicate against the M24 strain are plotted.
(TIF)

Figure S3 **Reversal of GIA activity using diverse allelic proteins.** Anti-3D7 or anti-QV serum pools were diluted to yield ∼60% inhibition of 3D7 parasite strain. Seven AMA1 allelic proteins (3D7, FVO, HB3, W2mef, 7G8, M24 and 102-1) were added to the invasion inhibition assay (2.8 µM or ∼150 µg/ml) to compete out the availability of cross-reacting antibodies. Bars are mean+s.e.m of three experiments. Percent reversal of inhibition = (inhibition in presence of *P. berghei* AMA1−inhibition in the presence of the test antigen)/inhibition in presence of *P. berghei* AMA1.
(TIF)

Figure S4 **GIA conducted by the NIH reference laboratory using QV, trivalent and bivalent vaccine-induced IgG.** (**A**) Total IgG at 2.5 mg/ml pooled from 3 rabbits vaccinated with QV or the two trivalent vaccines (3D7+FVO+W2mef and 3D7+FVO+HB3) or a bivalent vaccine (3D7+FVO). IgG against *P. berghei* AMA1 was used as the control. Lines are median inhibition across-strains. (**B**) Dose response of invasion inhibition by anti-QV IgG pools from two independently vaccinated groups of three rabbits. The concentration of total IgG that gave 50% invasion inhibition (IC_50_) against the 3D7 parasite strain was 0.16 and 0.19 mg/ml for QV pool-1 and QV pool-2 respectively.
(TIF)

Figure S5 **Chimeras used in GIA reversal assays and mapping of conformational mAb epitopes.** (**A**) Contiguous surface residues of *P. falciparum* 3D7 AMA1 (color) were grafted onto a scaffold of rodent malaria parasite *P. berghei* AMA1 (gray residues). *P. falciparum* AMA1 structural elements representing three domains as defined by the crystal structure (chimeras Cry D1, Cry D2, Cry D3), the polymorphic and conserved face (chimeras POLY and CONS), residues at the rim of the hydrophobic trough (HT) and the domain-2 loop together with the neighboring 1e-loop (chimera D2+1e) were displayed. Three linear domains as defined by the disulphide bonded pattern were also displayed (chimeras Lin D1, Lin D2, Lin D3, Lin D1+2 and Lin D2+3). (**B**) The genes for the chimeras were expressed and proteins were purified as shown on the non-reduced coomasie blue gel. The *P. falciparum* 3D7 AMA1 and *P. berghei* AMA1 proteins (3D7 and PbAMA) were also run on this gel. (**C**) Reversal of 3D7 parasite invasion inhibition, mediated by a pool of three 3D7 AMA1 vaccinated rabbit sera using 3D7 AMA1 based chimeras (CryD1, CryD2, CryD3, POLY and CONS), added individually or in combination, at 4 µM final concentration. Data are mean of three independent experiments.
(TIF)

Figure S6 **Dose response GIA.** Serial dilution of monoclonal antibodies were tested against the 3D7 parasite strain and 30% inhibitory mAb concentration (IC_30_) was calculated. Polyclonal anti-3D7 AMA1 IgG that was affinity purified over a 3D7 AMA1 affinity column was also tested. Results are from a single experiment.
(TIF)

Figure S7 **Binding of 1e-loop mAbs to phage-displayed mutant AMA1.** Residue 230 (within loop-1e) was switched from K to A, on a phage expressing the 3D7 AMA1 ectodomain. Binding of the mAbs against wild-type (wt) and mutant phage (K_230_A) was measured as OD_450_ (error bar is the range of duplicate wells). MAbs 5G8 (N-terminal pro-domain), 4G2 and 1F9 bind to regions outside the 1e-loop were used as negative controls.
(TIF)

Figure S8 **Region-specific ELISA.** Polyclonal serum affinity purified over an M24 affinity column was tested in the chimera ELISA. Region-specific titers (% of total) were calculated as the ratio of end-point titers against a 3D7 chimera relative to the end-point titer against 3D7 AMA1 protein.
(TIF)

Table S1 **The list of 201 isolates whose AMA1 sequences were used to create the dendrogram in** **Fig. 1A** **.** The strains highlighted in yellow were tested in invasion inhibition assays and found to be susceptible to QV antibodies. AMA1 field isolate sequences were obtained from Genbank [35], [43], [87], [88] and lab isolates sequences were obtained from either Genbank or the source laboratory.
(TIF)

Table S2 **Sequence of protein chimeras.** An alignment of *P. berghei* ANKA strain AMA1 is shown along with the residues that were switched to *P. falciparum* 3D7 sequence (boxed in gray). The boundaries of loops and domains are shown.
(DOCX)

## References

[1] Sachs J, Malaney P (2002) The economic and social burden of malaria. Nature 415: 680–685. 11832956 10.1038/415680a

[2] Crompton PD, Miura K, Traore B, Kayentao K, Ongoiba A, et al (2010) In vitro growth-inhibitory activity and malaria risk in a cohort study in mali. Infect Immun 78: 737–745. 19917712 10.1128/IAI.00960-09 PMC2812204

[3] Crompton PD, Pierce SK, Miller LH (2010) Advances and challenges in malaria vaccine development. J Clin Invest 120: 4168–4178. 21123952 10.1172/JCI44423 PMC2994342

[4] Tham WH, Healer J, Cowman AF (2012) Erythrocyte and reticulocyte binding-like proteins of Plasmodium falciparum. Trends Parasitol 28: 23–30. 22178537 10.1016/j.pt.2011.10.002

[5] Remarque EJ, Faber BW, Kocken CH, Thomas AW (2008) Apical membrane antigen 1: a malaria vaccine candidate in review. Trends Parasitol 24: 74–84. 18226584 10.1016/j.pt.2007.12.002

[6] Triglia T, Healer J, Caruana SR, Hodder AN, Anders RF, et al (2000) Apical membrane antigen 1 plays a central role in erythrocyte invasion by Plasmodium species. Mol Microbiol 38: 706–718. 11115107 10.1046/j.1365-2958.2000.02175.x

[7] Mahdi Abdel Hamid M, Remarque EJ, van Duivenvoorde LM, van der Werff N, Walraven V, et al (2011) Vaccination with Plasmodium knowlesi AMA1 formulated in the novel adjuvant co-vaccine HT protects against blood-stage challenge in rhesus macaques. PLoS One 6: e20547. 21655233 10.1371/journal.pone.0020547 PMC3105089

[8] Dutta S, Sullivan JS, Grady KK, Haynes JD, Komisar J, et al (2009) High antibody titer against apical membrane antigen-1 is required to protect against malaria in the Aotus model. PLoS One 4: e8138. 19997632 10.1371/journal.pone.0008138 PMC2780715

[9] Hodder AN, Crewther PE, Anders RF (2001) Specificity of the protective antibody response to apical membrane antigen 1. Infect Immun 69: 3286–3294. 11292751 10.1128/IAI.69.5.3286-3294.2001 PMC98287

[10] Polley SD, Mwangi T, Kocken CH, Thomas AW, Dutta S, et al (2004) Human antibodies to recombinant protein constructs of Plasmodium falciparum Apical Membrane Antigen 1 (AMA1) and their associations with protection from malaria. Vaccine 23: 718–728. 15542195 10.1016/j.vaccine.2004.05.031

[11] Stanisic DI, Richards JS, McCallum FJ, Michon P, King CL, et al (2009) Immunoglobulin G subclass-specific responses against Plasmodium falciparum merozoite antigens are associated with control of parasitemia and protection from symptomatic illness. Infect Immun 77: 1165–1174. 19139189 10.1128/IAI.01129-08 PMC2643653

[12] Coley AM, Parisi K, Masciantonio R, Hoeck J, Casey JL, et al (2006) The most polymorphic residue on Plasmodium falciparum apical membrane antigen 1 determines binding of an invasion-inhibitory antibody. Infect Immun 74: 2628–2636. 16622199 10.1128/IAI.74.5.2628-2636.2006 PMC1459722

[13] Crewther PE, Matthew ML, Flegg RH, Anders RF (1996) Protective immune responses to apical membrane antigen 1 of Plasmodium chabaudi involve recognition of strain-specific epitopes. Infect Immun 64: 3310–3317. 8757869 10.1128/iai.64.8.3310-3317.1996 PMC174223

[14] Healer J, Murphy V, Hodder AN, Masciantonio R, Gemmill AW, et al (2004) Allelic polymorphisms in apical membrane antigen-1 are responsible for evasion of antibody-mediated inhibition in Plasmodium falciparum. Mol Microbiol 52: 159–168. 15049818 10.1111/j.1365-2958.2003.03974.x

[15] Thera MA, Doumbo OK, Coulibaly D, Laurens MB, Ouattara A, et al (2011) A field trial to assess a blood-stage malaria vaccine. N Engl J Med 365: 1004–1013. 21916638 10.1056/NEJMoa1008115 PMC3242358

[16] Remarque EJ, Roestenberg M, Younis S, Walraven V, van der Werff N, et al (2012) Humoral immune responses to a single allele PfAMA1 vaccine in healthy malaria-naive adults. PLoS One 7: e38898. 22768052 10.1371/journal.pone.0038898 PMC3387192

[17] Dutta S, Lalitha PV, Ware LA, Barbosa A, Moch JK, et al (2002) Purification, characterization, and immunogenicity of the refolded ectodomain of the Plasmodium falciparum apical membrane antigen 1 expressed in Escherichia coli. Infect Immun 70: 3101–3110. 12011004 10.1128/IAI.70.6.3101-3110.2002 PMC127972

[18] Takala SL, Drissa C, Thera MA, Batchelor AH, Cummings MP, Ananias AA, Ouattara A, Niangaly A, Djimde AA, Plowe CV (2009) Extreme Polymorphism in a Vaccine antigen and risk of clinical malaria: Implications for vaccine development. Science Translational Med 1: 10. 10.1126/scitranslmed.3000257 PMC2822345 20165550

[19] Kennedy MC, Wang J, Zhang Y, Miles AP, Chitsaz F, et al (2002) In vitro studies with recombinant Plasmodium falciparum apical membrane antigen 1 (AMA1): production and activity of an AMA1 vaccine and generation of a multiallelic response. Infect Immun 70: 6948–6960. 12438374 10.1128/IAI.70.12.6948-6960.2002 PMC133034

[20] Sagara I, Dicko A, Ellis RD, Fay MP, Diawara SI, et al (2009) A randomized controlled phase 2 trial of the blood stage AMA1-C1/Alhydrogel malaria vaccine in children in Mali. Vaccine 27: 3090–3098. 19428923 10.1016/j.vaccine.2009.03.014 PMC2713037

[21] Narum DL, Thomas AW (1994) Differential localization of full-length and processed forms of PF83/AMA-1 an apical membrane antigen of Plasmodium falciparum merozoites. Mol Biochem Parasitol 67: 59–68. 7838184 10.1016/0166-6851(94)90096-5

[22] Howell SA, Withers-Martinez C, Kocken CH, Thomas AW, Blackman MJ (2001) Proteolytic processing and primary structure of Plasmodium falciparum apical membrane antigen-1. J Biol Chem 276: 31311–31320. 11399764 10.1074/jbc.M103076200

[23] Howell SA, Well I, Fleck SL, Kettleborough C, Collins CR, et al (2003) A single malaria merozoite serine protease mediates shedding of multiple surface proteins by juxtamembrane cleavage. J Biol Chem 278: 23890–23898. 12686561 10.1074/jbc.M302160200

[24] Cao J, Kaneko O, Thongkukiatkul A, Tachibana M, Otsuki H, et al (2009) Rhoptry neck protein RON2 forms a complex with microneme protein AMA1 in Plasmodium falciparum merozoites. Parasitol Int 58: 29–35. 18952195 10.1016/j.parint.2008.09.005

[25] Tonkin ML, Roques M, Lamarque MH, Pugniere M, Douguet D, et al (2011) Host cell invasion by apicomplexan parasites: insights from the co-structure of AMA1 with a RON2 peptide. Science 333: 463–467. 21778402 10.1126/science.1204988

[26] Richard D, MacRaild CA, Riglar DT, Chan JA, Foley M, et al (2010) Interaction between Plasmodium falciparum apical membrane antigen 1 and the rhoptry neck protein complex defines a key step in the erythrocyte invasion process of malaria parasites. J Biol Chem 285: 14815–14822. 20228060 10.1074/jbc.M109.080770 PMC2863225

[27] Baum J, Cowman AF (2011) Biochemistry. Revealing a parasite's invasive trick. Science 333: 410–411. 21778386 10.1126/science.1209875

[28] Dutta S, Lee SY, Batchelor AH, Lanar DE (2007) Structural basis of antigenic escape of a malaria vaccine candidate. Proc Natl Acad Sci U S A 104: 12488–12493. 17636123 10.1073/pnas.0701464104 PMC1941496

[29] Coley AM, Gupta A, Murphy VJ, Bai T, Kim H, et al (2007) Structure of the malaria antigen AMA1 in complex with a growth-inhibitory antibody. PLoS Pathog 3: 1308–1319. 17907804 10.1371/journal.ppat.0030138 PMC2323298

[30] Kocken CH, van der Wel AM, Dubbeld MA, Narum DL, van de Rijke FM, et al (1998) Precise timing of expression of a Plasmodium falciparum-derived transgene in Plasmodium berghei is a critical determinant of subsequent subcellular localization. J Biol Chem 273: 15119–15124. 9614123 10.1074/jbc.273.24.15119

[31] Collins CR, Withers-Martinez C, Bentley GA, Batchelor AH, Thomas AW, et al (2007) Fine mapping of an epitope recognized by an invasion-inhibitory monoclonal antibody on the malaria vaccine candidate apical membrane antigen 1. J Biol Chem 282: 7431–7441. 17192270 10.1074/jbc.M610562200

[32] Bai T, Becker M, Gupta A, Strike P, Murphy VJ, et al (2005) Structure of AMA1 from Plasmodium falciparum reveals a clustering of polymorphisms that surround a conserved hydrophobic pocket. Proc Natl Acad Sci U S A 102: 12736–12741. 16129835 10.1073/pnas.0501808102 PMC1200259

[33] Pizarro JC, Vulliez-Le Normand B, Chesne-Seck ML, Collins CR, Withers-Martinez C, et al (2005) Crystal structure of the malaria vaccine candidate apical membrane antigen 1. Science 308: 408–411. 15731407 10.1126/science.1107449

[34] Dutta S, Haynes JD, Barbosa A, Ware LA, Snavely JD, et al (2005) Mode of action of invasion-inhibitory antibodies directed against apical membrane antigen 1 of Plasmodium falciparum. Infect Immun 73: 2116–2122. 15784553 10.1128/IAI.73.4.2116-2122.2005 PMC1087451

[35] Polley SD, Conway DJ (2001) Strong diversifying selection on domains of the Plasmodium falciparum apical membrane antigen 1 gene. Genetics 158: 1505–1512. 11514442 10.1093/genetics/158.4.1505 PMC1461755

[36] Duan J, Mu J, Thera MA, Joy D, Kosakovsky Pond SL, et al (2008) Population structure of the genes encoding the polymorphic Plasmodium falciparum apical membrane antigen 1: implications for vaccine design. Proc Natl Acad Sci U S A 105: 7857–7862. 18515425 10.1073/pnas.0802328105 PMC2409418

[37] Miura K, Herrera R, Diouf A, Zhou H, Mu J, et al (2013) Overcoming allelic specificity by immunization with five allelic forms of Plasmodium falciparum apical membrane antigen 1. Infect Immun 81: 1491–1501. 23429537 10.1128/IAI.01414-12 PMC3648006

[38] Remarque EJ, Faber BW, Kocken CH, Thomas AW (2008) A diversity-covering approach to immunization with Plasmodium falciparum apical membrane antigen 1 induces broader allelic recognition and growth inhibition responses in rabbits. Infect Immun 76: 2660–2670. 18378635 10.1128/IAI.00170-08 PMC2423090

[39] Kusi KA, Remarque EJ, Riasat V, Walraven V, Thomas AW, et al (2011) Safety and immunogenicity of multi-antigen AMA1-based vaccines formulated with CoVaccine HT and Montanide ISA 51 in rhesus macaques. Malar J 10: 182. 21726452 10.1186/1475-2875-10-182 PMC3142537

[40] Ouattara A, Takala-Harrison S, Thera MA, Coulibaly D, Niangaly A, et al (2013) Molecular basis of allele-specific efficacy of a blood-stage malaria vaccine: vaccine development implications. J Infect Dis 207: 511–519. 23204168 10.1093/infdis/jis709 PMC3537449

[41] Kusi KA, Faber BW, Thomas AW, Remarque EJ (2009) Humoral immune response to mixed PfAMA1 alleles; multivalent PfAMA1 vaccines induce broad specificity. PLoS One 4: e8110. 19956619 10.1371/journal.pone.0008110 PMC2779588

[42] Kusi KA, Faber BW, van der Eijk M, Thomas AW, Kocken CH, et al (2011) Immunization with different PfAMA1 alleles in sequence induces clonal imprint humoral responses that are similar to responses induced by the same alleles as a vaccine cocktail in rabbits. Malar J 10: 40. 21320299 10.1186/1475-2875-10-40 PMC3050776

[43] Drew DR, Hodder AN, Wilson DW, Foley M, Mueller I, et al (2012) Defining the Antigenic Diversity of Plasmodium falciparum Apical Membrane Antigen 1 and the Requirements for a Multi-Allele Vaccine against Malaria. PLoS One 7: e51023. 23227229 10.1371/journal.pone.0051023 PMC3515520

[44] Haynes JD, Moch JK, Smoot DS (2002) Erythrocytic malaria growth or invasion inhibition assays with emphasis on suspension culture GIA. Methods Mol Med 72: 535–554. 12125152 10.1385/1-59259-271-6:535

[45] Malkin EM, Diemert DJ, McArthur JH, Perreault JR, Miles AP, et al (2005) Phase 1 clinical trial of apical membrane antigen 1: an asexual blood-stage vaccine for Plasmodium falciparum malaria. Infect Immun 73: 3677–3685. 15908397 10.1128/IAI.73.6.3677-3685.2005 PMC1111886

[46] Persson KE, Lee CT, Marsh K, Beeson JG (2006) Development and optimization of high-throughput methods to measure Plasmodium falciparum-specific growth inhibitory antibodies. J Clin Microbiol 44: 1665–1673. 16672391 10.1128/JCM.44.5.1665-1673.2006 PMC1479166

[47] Chesne-Seck ML, Pizarro JC, Vulliez-Le Normand B, Collins CR, Blackman MJ, et al (2005) Structural comparison of apical membrane antigen 1 orthologues and paralogues in apicomplexan parasites. Mol Biochem Parasitol 144: 55–67. 16154214 10.1016/j.molbiopara.2005.07.007

[48] Crawford J, Tonkin ML, Grujic O, Boulanger MJ (2010) Structural characterization of apical membrane antigen 1 (AMA1) from Toxoplasma gondii. J Biol Chem 285: 15644–15652. 20304917 10.1074/jbc.M109.092619 PMC2865318

[49] Nair M, Hinds MG, Coley AM, Hodder AN, Foley M, et al (2002) Structure of domain III of the blood-stage malaria vaccine candidate, Plasmodium falciparum apical membrane antigen 1 (AMA1). J Mol Biol 322: 741–753. 12270711 10.1016/s0022-2836(02)00806-9

[50] Nair M, Hodder AN, Hinds MG, Anders RF, Norton RS (2001) Assignment of 1H, 13C and 15N resonances of domain III of the ectodomain of apical membrane antigen 1 from Plasmodium falciparum. J Biomol NMR 19: 85–86. 11246861 10.1023/a:1008342111585

[51] Hodder AN, Crewther PE, Matthew ML, Reid GE, Moritz RL, et al (1996) The disulfide bond structure of Plasmodium apical membrane antigen-1. J Biol Chem 271: 29446–29452. 8910611 10.1074/jbc.271.46.29446

[52] Collins CR, Withers-Martinez C, Hackett F, Blackman MJ (2009) An inhibitory antibody blocks interactions between components of the malarial invasion machinery. PLoS Pathog 5: e1000273. 19165323 10.1371/journal.ppat.1000273 PMC2621342

[53] Woehlbier U, Epp C, Hackett F, Blackman MJ, Bujard H (2010) Antibodies against multiple merozoite surface antigens of the human malaria parasite Plasmodium falciparum inhibit parasite maturation and red blood cell invasion. Malar J 9: 77. 20298576 10.1186/1475-2875-9-77 PMC2847572

[54] Howell SA, Hackett F, Jongco AM, Withers-Martinez C, Kim K, et al (2005) Distinct mechanisms govern proteolytic shedding of a key invasion protein in apicomplexan pathogens. Mol Microbiol 57: 1342–1356. 16102004 10.1111/j.1365-2958.2005.04772.x

[55] Williams AR, Douglas AD, Miura K, Illingworth JJ, Choudhary P, et al (2012) Enhancing blockade of Plasmodium falciparum erythrocyte invasion: assessing combinations of antibodies against PfRH5 and other merozoite antigens. PLoS Pathog 8: e1002991. 23144611 10.1371/journal.ppat.1002991 PMC3493472

[56] Bliss CI (1939) The Toxicity of Poisons Applied Jointly. Annals of Applied Biology 26: 25.

[57] Lalitha PV, Ware LA, Barbosa A, Dutta S, Moch JK, et al (2004) Production of the subdomains of the Plasmodium falciparum apical membrane antigen 1 ectodomain and analysis of the immune response. Infect Immun 72: 4464–4470. 15271904 10.1128/IAI.72.8.4464-4470.2004 PMC470679

[58] Ntumngia FB, Schloegel J, McHenry AM, Barnes SJ, George MT, et al (2013) Immunogenicity of single versus mixed allele vaccines of Plasmodium vivax Duffy binding protein region II. Vaccine 31: 4382–4388. 23916294 10.1016/j.vaccine.2013.07.002 PMC4497540

[59] Li GM, Chiu C, Wrammert J, McCausland M, Andrews SF, et al (2012) Pandemic H1N1 influenza vaccine induces a recall response in humans that favors broadly cross-reactive memory B cells. Proc Natl Acad Sci U S A 109: 9047–9052. 22615367 10.1073/pnas.1118979109 PMC3384143

[60] Pica N, Hai R, Krammer F, Wang TT, Maamary J, et al (2012) Hemagglutinin stalk antibodies elicited by the 2009 pandemic influenza virus as a mechanism for the extinction of seasonal H1N1 viruses. Proc Natl Acad Sci U S A 109: 2573–2578. 22308500 10.1073/pnas.1200039109 PMC3289326

[61] Krammer F, Pica N, Hai R, Tan GS, Palese P (2012) Hemagglutinin Stalk-Reactive Antibodies Are Boosted following Sequential Infection with Seasonal and Pandemic H1N1 Influenza Virus in Mice. J Virol 86: 10302–10307. 22787225 10.1128/JVI.01336-12 PMC3457330

[62] Miller MS, Tsibane T, Krammer F, Hai R, Rahmat S, et al (2012) 1976 and 2009 H1N1 Influenza Virus Vaccines Boost Anti-Hemagglutinin Stalk Antibodies in Humans. J Infect Dis 207: 98–105. 23087428 10.1093/infdis/jis652 PMC3523798

[63] Kusi KA, Dodoo D, Bosomprah S, van der Eijk M, Faber BW, et al (2012) Measurement of the plasma levels of antibodies against the polymorphic vaccine candidate apical membrane antigen 1 in a malaria-exposed population. BMC Infect Dis 12: 32. 22299616 10.1186/1471-2334-12-32 PMC3317819

[64] Cortes A, Mellombo M, Masciantonio R, Murphy VJ, Reeder JC, et al (2005) Allele specificity of naturally acquired antibody responses against Plasmodium falciparum apical membrane antigen 1. Infect Immun 73: 422–430. 15618180 10.1128/IAI.73.1.422-430.2005 PMC538974

[65] Vulliez-Le Normand B, Tonkin ML, Lamarque MH, Langer S, Hoos S, et al (2012) Structural and functional insights into the malaria parasite moving junction complex. PLoS Pathog 8: e1002755. 22737069 10.1371/journal.ppat.1002755 PMC3380929

[66] Mueller MS, Renard A, Boato F, Vogel D, Naegeli M, et al (2003) Induction of parasite growth-inhibitory antibodies by a virosomal formulation of a peptidomimetic of loop I from domain III of Plasmodium falciparum apical membrane antigen 1. Infect Immun 71: 4749–4758. 12874357 10.1128/IAI.71.8.4749-4758.2003 PMC166038

[67] Olivieri A, Collins CR, Hackett F, Withers-Martinez C, Marshall J, et al (2011) Juxtamembrane shedding of Plasmodium falciparum AMA1 is sequence independent and essential, and helps evade invasion-inhibitory antibodies. PLoS Pathog 7: e1002448. 22194692 10.1371/journal.ppat.1002448 PMC3240622

[68] Doria-Rose NA, Louder MK, Yang Z, O'Dell S, Nason M, et al (2012) HIV-1 neutralization coverage is improved by combining monoclonal antibodies that target independent epitopes. J Virol 86: 3393–3397. 22258252 10.1128/JVI.06745-11 PMC3302320

[69] Miura K, Zhou H, Muratova OV, Orcutt AC, Giersing B, et al (2007) In immunization with Plasmodium falciparum apical membrane antigen 1, the specificity of antibodies depends on the species immunized. Infect Immun 75: 5827–5836. 17923516 10.1128/IAI.00593-07 PMC2168362

[70] Faber BW, Younis S, Remarque EJ, Rodriguez Garcia R, Riasat V, et al (2013) Diversity covering AMA1-MSP119 fusion proteins as malaria vaccines. Infect Immun 81: 1479–1490. 23429538 10.1128/IAI.01267-12 PMC3648017

[71] Dutta S, Dlugosz LS, Clayton JW, Pool CD, Haynes JD, et al (2010) Alanine mutagenesis of the primary antigenic escape residue cluster, c1, of apical membrane antigen 1. Infect Immun 78: 661–671. 19948834 10.1128/IAI.00866-09 PMC2812209

[72] Kusi KA, Faber BW, Riasat V, Thomas AW, Kocken CH, et al (2010) Generation of humoral immune responses to multi-allele PfAMA1 vaccines; effect of adjuvant and number of component alleles on the breadth of response. PLoS One 5: e15391. 21082025 10.1371/journal.pone.0015391 PMC2972715

[73] Douglas AD, Williams AR, Illingworth JJ, Kamuyu G, Biswas S, et al (2011) The blood-stage malaria antigen PfRH5 is susceptible to vaccine-inducible cross-strain neutralizing antibody. Nat Commun 2: 601. 22186897 10.1038/ncomms1615 PMC3504505

[74] Polhemus ME, Magill AJ, Cummings JF, Kester KE, Ockenhouse CF, et al (2007) Phase I dose escalation safety and immunogenicity trial of Plasmodium falciparum apical membrane protein (AMA-1) FMP2.1, adjuvanted with AS02A, in malaria-naive adults at the Walter Reed Army Institute of Research. Vaccine 25: 4203–4212. 17442466 10.1016/j.vaccine.2007.03.012

[75] Spring MD, Cummings JF, Ockenhouse CF, Dutta S, Reidler R, et al (2009) Phase 1/2a study of the malaria vaccine candidate apical membrane antigen-1 (AMA-1) administered in adjuvant system AS01B or AS02A. PLoS One 4: e5254. 19390585 10.1371/journal.pone.0005254 PMC2669163

[76] Bruder JT, Stefaniak ME, Patterson NB, Chen P, Konovalova S, et al (2010) Adenovectors induce functional antibodies capable of potent inhibition of blood stage malaria parasite growth. Vaccine 28: 3201–3210. 20188680 10.1016/j.vaccine.2010.02.024

[77] Sedegah M, Tamminga C, McGrath S, House B, Ganeshan H, et al (2011) Adenovirus 5-vectored P. falciparum vaccine expressing CSP and AMA1. Part A: safety and immunogenicity in seronegative adults. PLoS One 6: e24586. 22003383 10.1371/journal.pone.0024586 PMC3189181

[78] Cox NJ, Subbarao K (2000) Global epidemiology of influenza: past and present. Annu Rev Med 51: 407–421. 10774473 10.1146/annurev.med.51.1.407

[79] Polley SD, Chokejindachai W, Conway DJ (2003) Allele frequency-based analyses robustly map sequence sites under balancing selection in a malaria vaccine candidate antigen. Genetics 165: 555–561. 14573469 10.1093/genetics/165.2.555 PMC1462796

[80] Yoshiyama H, Mo H, Moore JP, Ho DD (1994) Characterization of mutants of human immunodeficiency virus type 1 that have escaped neutralization by a monoclonal antibody to the gp120 V2 loop. J Virol 68: 974–978. 7507188 10.1128/jvi.68.2.974-978.1994 PMC236535

[81] Moore PL, Gray ES, Wibmer CK, Bhiman JN, Nonyane M, et al (2012) Evolution of an HIV glycan-dependent broadly neutralizing antibody epitope through immune escape. Nat Med 18: 1688–1692. 23086475 10.1038/nm.2985 PMC3494733

[82] Barclay VC, Sim D, Chan BH, Nell LA, Rabaa MA, et al (2012) The evolutionary consequences of blood-stage vaccination on the rodent malaria Plasmodium chabaudi. PLoS Biol 10: e1001368. 22870063 10.1371/journal.pbio.1001368 PMC3409122

[83] Duncan CJ, Draper SJ (2012) Controlled human blood stage malaria infection: current status and potential applications. Am J Trop Med Hyg 86: 561–565. 22492136 10.4269/ajtmh.2012.11-0504 PMC3403771

[84] Pichyangkul S, Tongtawe P, Kum-Arb U, Yongvanitchit K, Gettayacamin M, et al (2009) Evaluation of the safety and immunogenicity of Plasmodium falciparum apical membrane antigen 1, merozoite surface protein 1 or RTS,S vaccines with adjuvant system AS02A administered alone or concurrently in rhesus monkeys. Vaccine 28: 452–462. 19857448 10.1016/j.vaccine.2009.10.022

[85] Wilson DW, Crabb BS, Beeson JG (2010) Development of fluorescent Plasmodium falciparum for in vitro growth inhibition assays. Malar J 9: 152. 20525251 10.1186/1475-2875-9-152 PMC2891815

[86] Dutta S, Haynes JD, Moch JK, Barbosa A, Lanar DE (2003) Invasion-inhibitory antibodies inhibit proteolytic processing of apical membrane antigen 1 of Plasmodium falciparum merozoites. Proc Natl Acad Sci U S A 100: 12295–12300. 14526103 10.1073/pnas.2032858100 PMC218752

[87] Escalante AA, Grebert HM, Chaiyaroj SC, Magris M, Biswas S, et al (2001) Polymorphism in the gene encoding the apical membrane antigen-1 (AMA-1) of Plasmodium falciparum. X. Asembo Bay Cohort Project. Mol Biochem Parasitol 113: 279–287. 11295182 10.1016/s0166-6851(01)00229-8

[88] Kocken CH, Narum DL, Massougbodji A, Ayivi B, Dubbeld MA, et al (2000) Molecular characterisation of Plasmodium reichenowi apical membrane antigen-1 (AMA-1), comparison with P. falciparum AMA-1, and antibody-mediated inhibition of red cell invasion. Mol Biochem Parasitol 109: 147–156. 10960173 10.1016/s0166-6851(00)00250-4

