# Correction: Overcoming Antigenic Diversity by Enhancing the Immunogenicity of Conserved Epitopes on the Malaria Vaccine Candidate Apical Membrane Antigen-1

**DOI:** 10.1371/annotation/be0d67d1-314f-4654-8a2f-b668d1e32bbd

**Published:** 2014-01-24

**Authors:** Sheetij Dutta, Lisa S. Dlugosz, Damien R. Drew, Xiaopeng Ge, Diouf Ababacar, Yazmin I. Rovira, J. Kathleen Moch, Meng Shi, Carole A. Long, Michael Foley, James G. Beeson, Robin F. Anders, Kazutoyo Miura, J. David Haynes, Adrian H. Batchelor

There is an error in the spelling of the first name of the author listed as Xiopeng Ge. The correct spelling is Xiaopeng Ge. 

