# Supplementary figures and images for: Overcoming Antigenic Diversity by Enhancing the Immunogenicity of Conserved Epitopes on the Malaria Vaccine Candidate Apical Membrane Antigen-1
Source: PLoS Pathog. 2013 Dec 26;9(12):e1003840. doi: 10.1371/journal.ppat.1003840 (PMC3873463; doi:10.1371/journal.ppat.1003840)

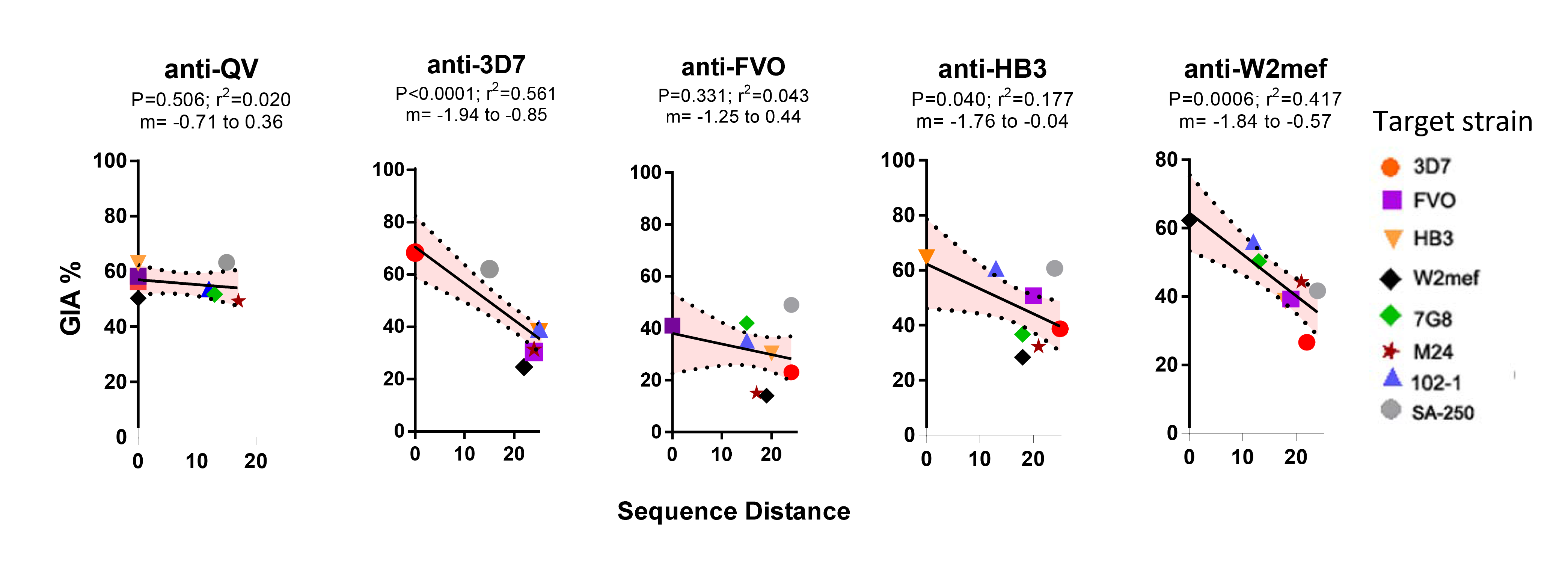

Supplement: Figure S1 — Inverse relationship between sequence distance and GIA. Mean invasion inhibition data from 3 rabbits was plotted against the amino acid difference between the vaccine and each of the 8 target strains (sequence distance), tested by the WRAIR GIA method. For anti-QV, the amino acid difference of a heterologous strain was against the most similar QV allele. Lines of best fit (solid black), error range (dotted black and pink), P value indicating if the slope is significantly non-zero, R2 and 95% CI of the slope (m) are shown. (TIF) [file ppat.1003840.s001.tif]

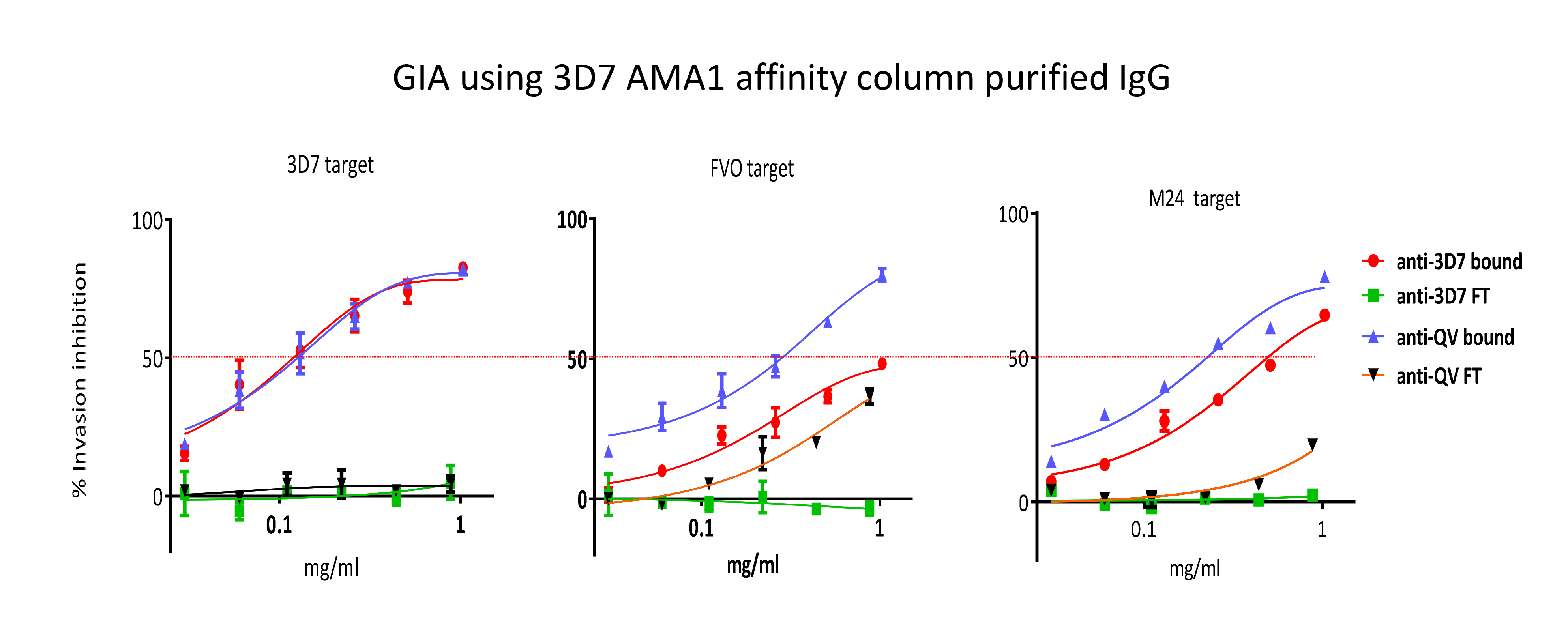

Supplement: Figure S2 — GIA with affinity purified antibodies used to calculate the IC50. Anti-3D7 and anti-Quadvax IgG were affinity purified over a 3D7 AMA1 column. Bound/eluted (bound; red and blue lines) or the flow-through fractions (FT; orange and green lines) were adjusted to equivalent IgG concentration and tested against 3D7, FVO and M24 parasite strains. Mean+s.e.m. of 3 independent experiments against 3D7 and FVO strains and one experiment in triplicate against the M24 strain are plotted. (TIF) [file ppat.1003840.s002.tif]

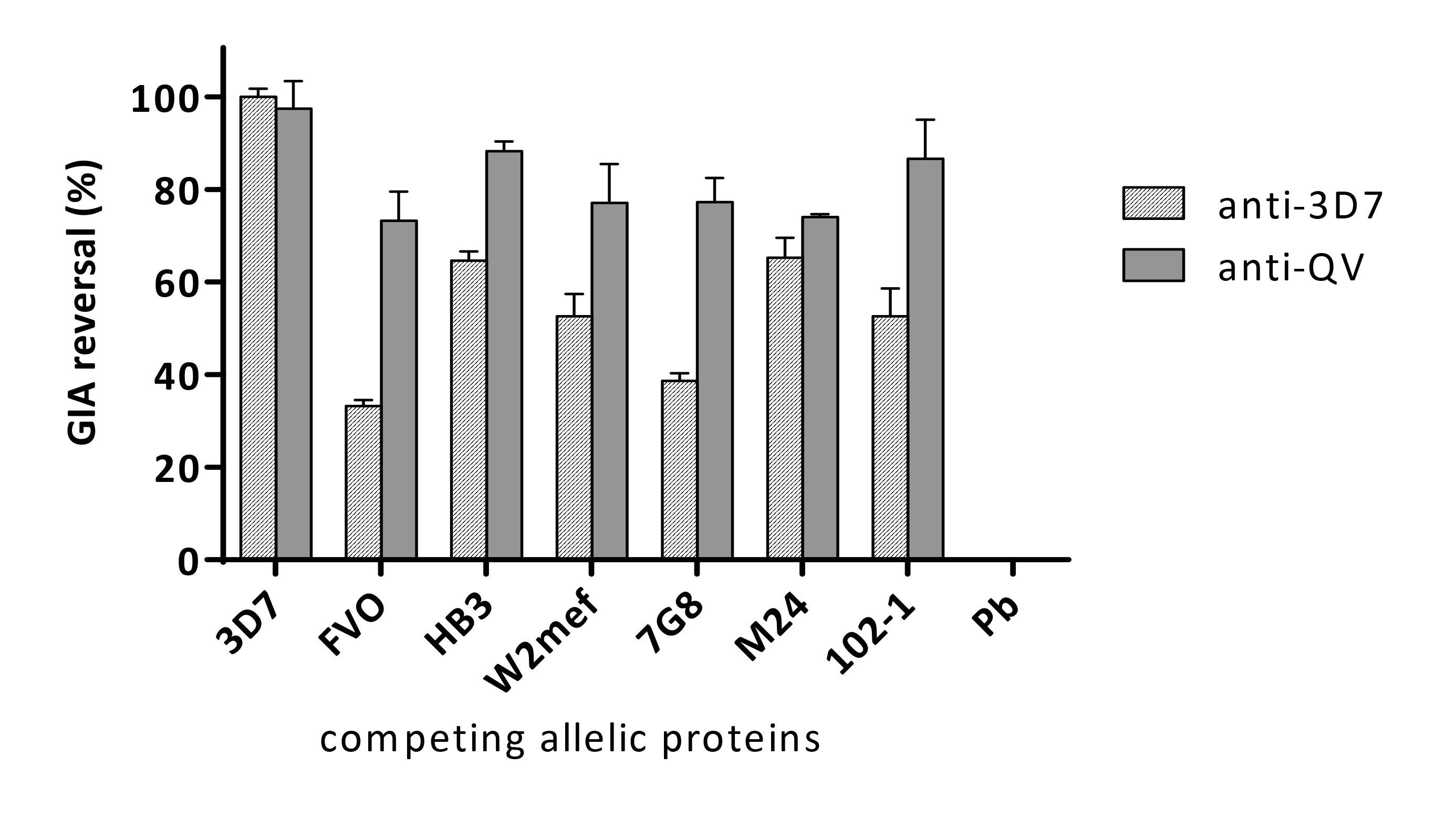

Supplement: Figure S3 — Reversal of GIA activity using diverse allelic proteins. Anti-3D7 or anti-QV serum pools were diluted to yield ∼60% inhibition of 3D7 parasite strain. Seven AMA1 allelic proteins (3D7, FVO, HB3, W2mef, 7G8, M24 and 102-1) were added to the invasion inhibition assay (2.8 µM or ∼150 µg/ml) to compete out the availability of cross-reacting antibodies. Bars are mean+s.e.m of three experiments. Percent reversal of inhibition = (inhibition in presence of P. berghei AMA1−inhibition in the presence of the test antigen)/inhibition in presence of P. berghei AMA1. (TIF) [file ppat.1003840.s003.tif]

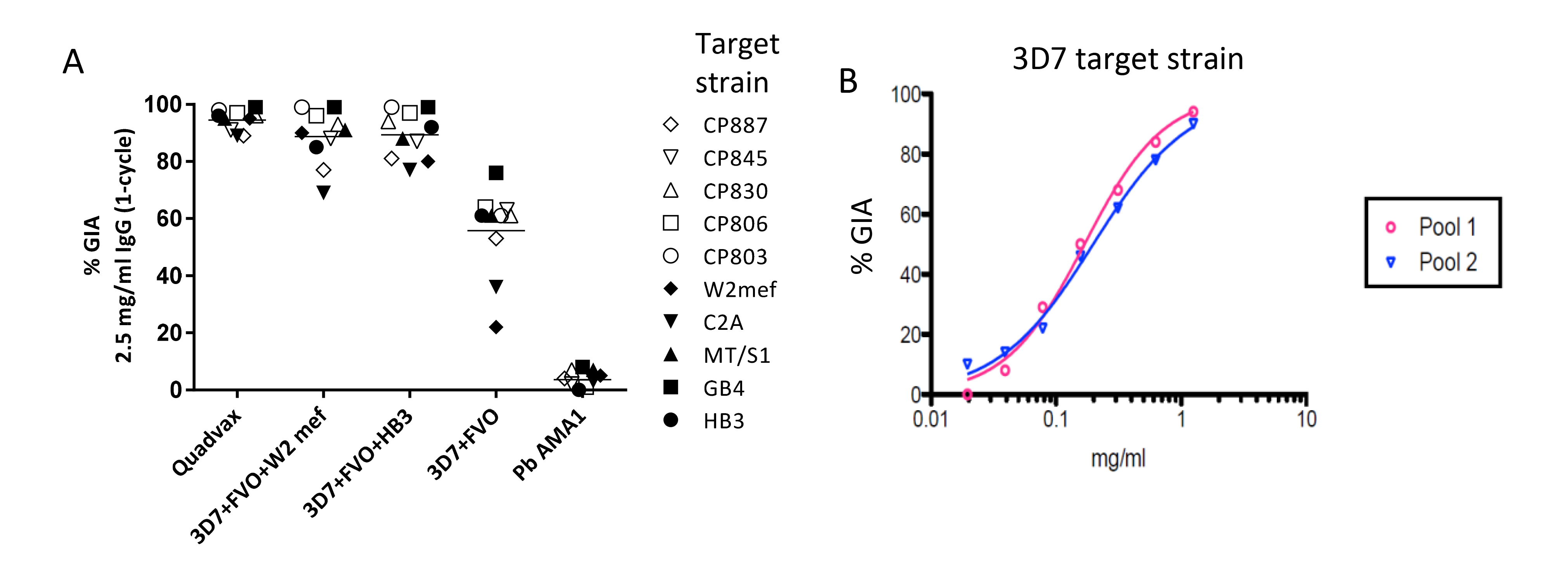

Supplement: Figure S4 — GIA conducted by the NIH reference laboratory using QV, trivalent and bivalent vaccine-induced IgG. (A) Total IgG at 2.5 mg/ml pooled from 3 rabbits vaccinated with QV or the two trivalent vaccines (3D7+FVO+W2mef and 3D7+FVO+HB3) or a bivalent vaccine (3D7+FVO). IgG against P. berghei AMA1 was used as the control. Lines are median inhibition across-strains. (B) Dose response of invasion inhibition by anti-QV IgG pools from two independently vaccinated groups of three rabbits. The concentration of total IgG that gave 50% invasion inhibition (IC50) against the 3D7 parasite strain was 0.16 and 0.19 mg/ml for QV pool-1 and QV pool-2 respectively. (TIF) [file ppat.1003840.s004.tif]

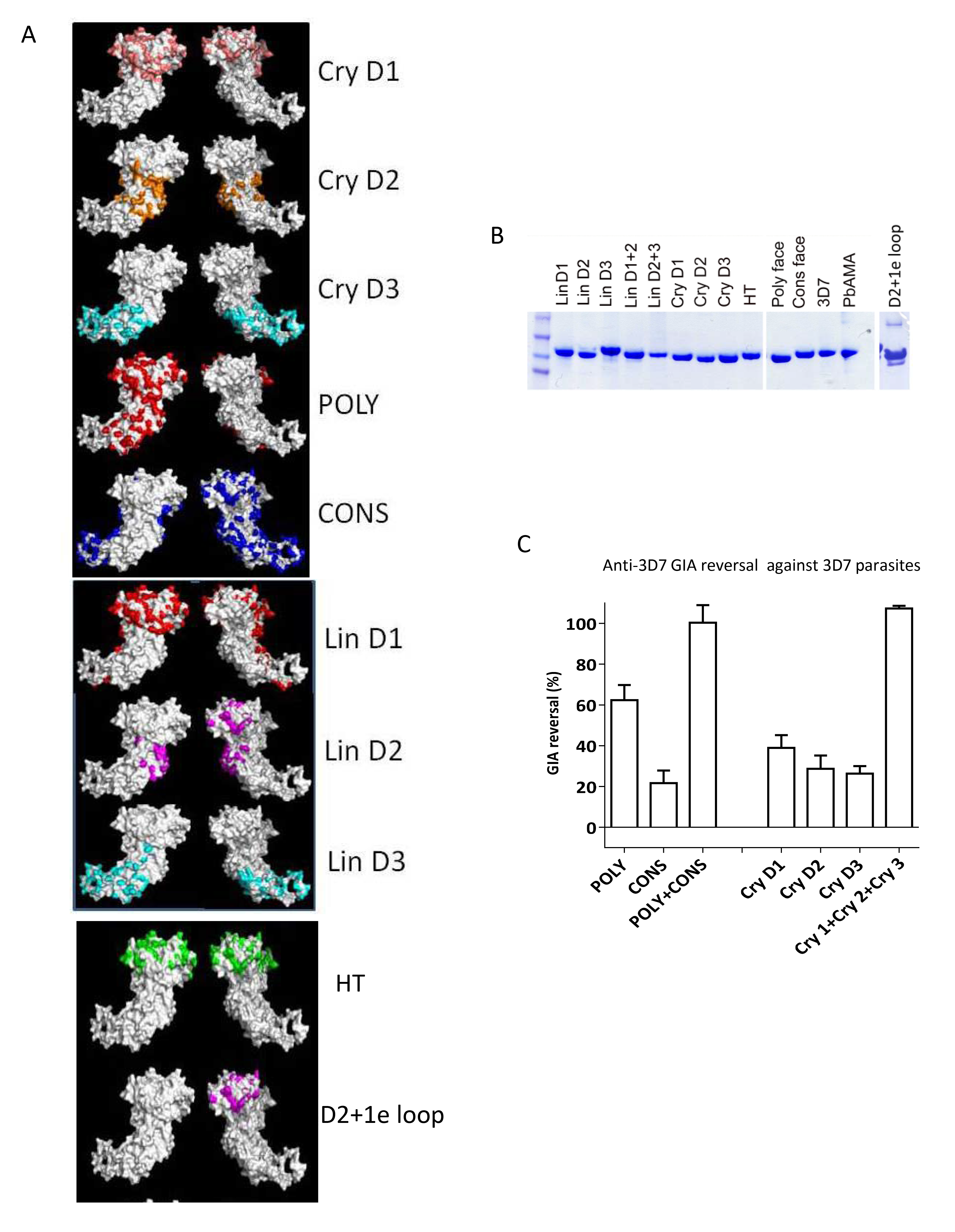

Supplement: Figure S5 — Chimeras used in GIA reversal assays and mapping of conformational mAb epitopes. (A) Contiguous surface residues of P. falciparum 3D7 AMA1 (color) were grafted onto a scaffold of rodent malaria parasite P. berghei AMA1 (gray residues). P. falciparum AMA1 structural elements representing three domains as defined by the crystal structure (chimeras Cry D1, Cry D2, Cry D3), the polymorphic and conserved face (chimeras POLY and CONS), residues at the rim of the hydrophobic trough (HT) and the domain-2 loop together with the neighboring 1e-loop (chimera D2+1e) were displayed. Three linear domains as defined by the disulphide bonded pattern were also displayed (chimeras Lin D1, Lin D2, Lin D3, Lin D1+2 and Lin D2+3). (B) The genes for the chimeras were expressed and proteins were purified as shown on the non-reduced coomasie blue gel. The P. falciparum 3D7 AMA1 and P. berghei AMA1 proteins (3D7 and PbAMA) were also run on this gel. (C) Reversal of 3D7 parasite invasion inhibition, mediated by a pool of three 3D7 AMA1 vaccinated rabbit sera using 3D7 AMA1 based chimeras (CryD1, CryD2, CryD3, POLY and CONS), added individually or in combination, at 4 µM final concentration. Data are mean of three independent experiments. (TIF) [file ppat.1003840.s005.tif]

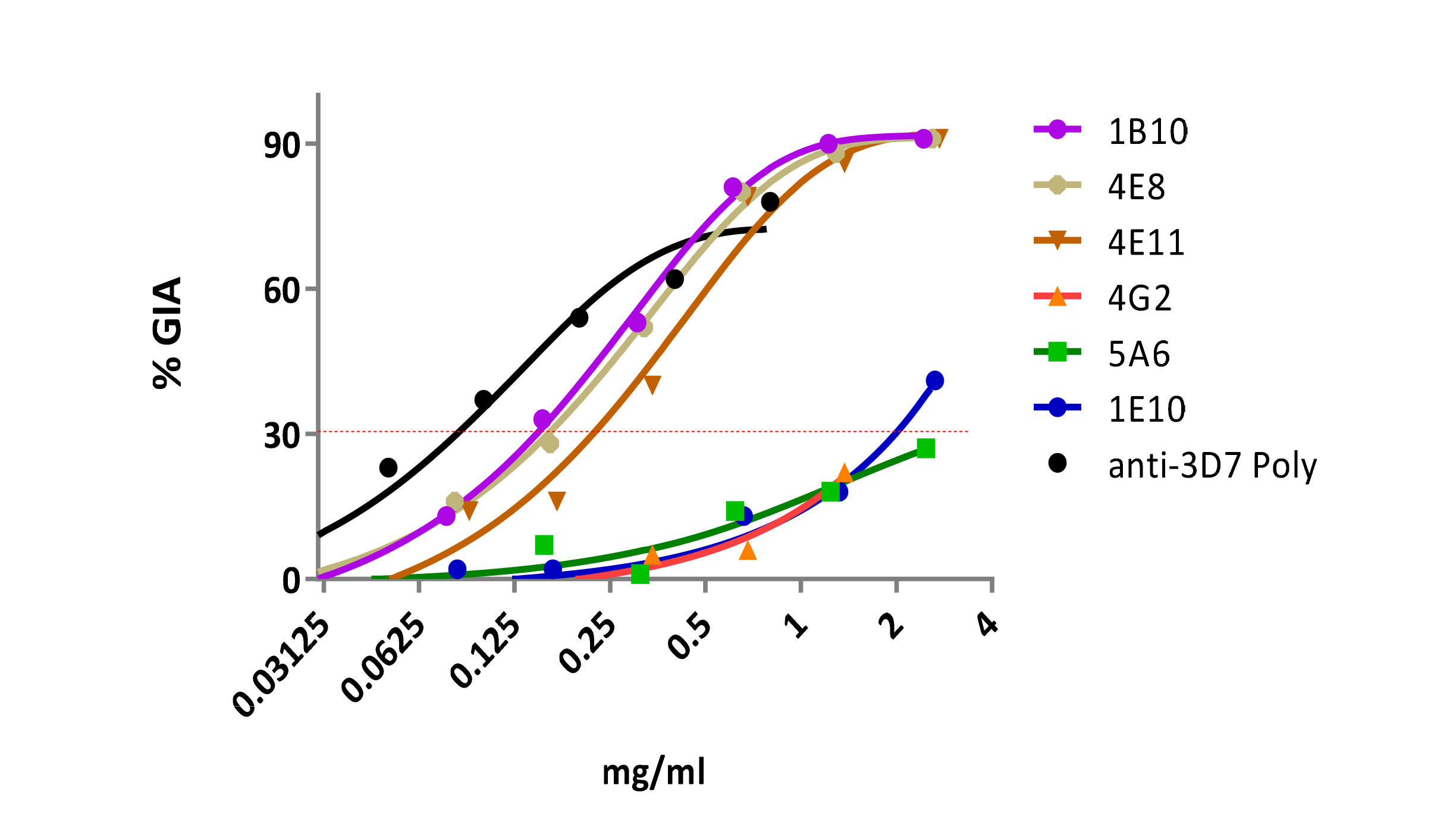

Supplement: Figure S6 — Dose response GIA. Serial dilution of monoclonal antibodies were tested against the 3D7 parasite strain and 30% inhibitory mAb concentration (IC30) was calculated. Polyclonal anti-3D7 AMA1 IgG that was affinity purified over a 3D7 AMA1 affinity column was also tested. Results are from a single experiment. (TIF) [file ppat.1003840.s006.tif]

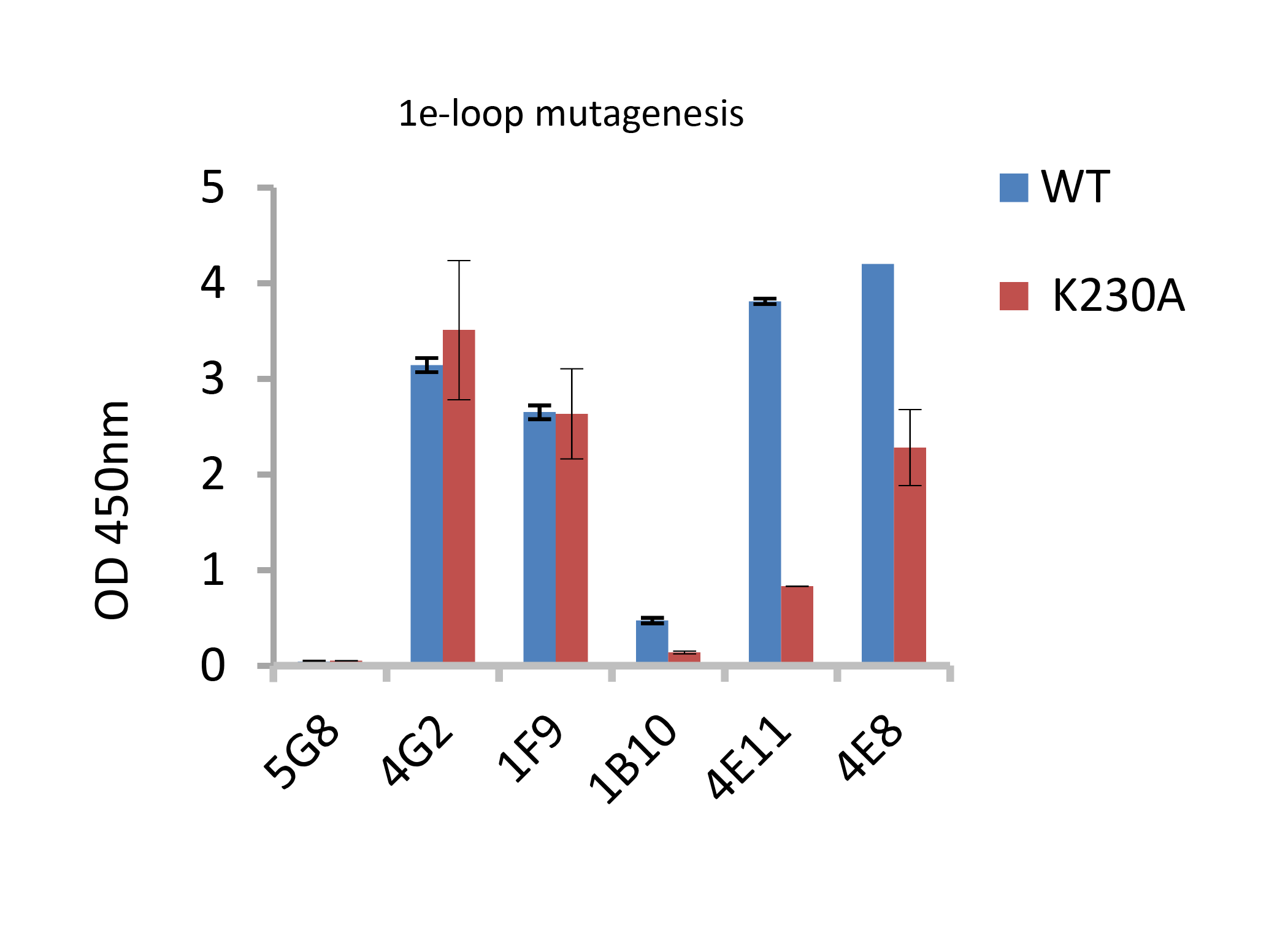

Supplement: Figure S7 — Binding of 1e-loop mAbs to phage-displayed mutant AMA1. Residue 230 (within loop-1e) was switched from K to A, on a phage expressing the 3D7 AMA1 ectodomain. Binding of the mAbs against wild-type (wt) and mutant phage (K230A) was measured as OD450 (error bar is the range of duplicate wells). MAbs 5G8 (N-terminal pro-domain), 4G2 and 1F9 bind to regions outside the 1e-loop were used as negative controls. (TIF) [file ppat.1003840.s007.tif]

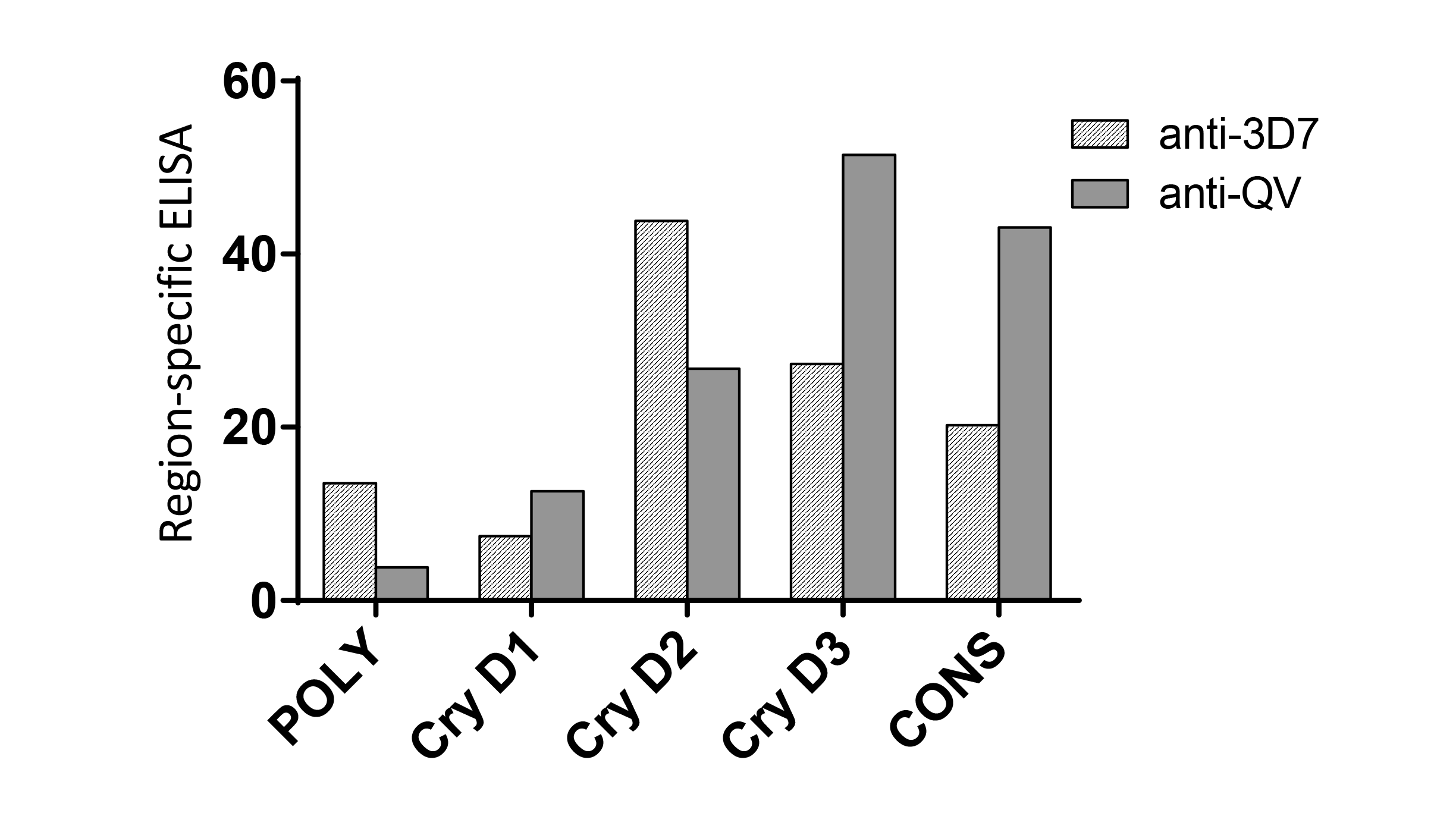

Supplement: Figure S8 — Region-specific ELISA. Polyclonal serum affinity purified over an M24 affinity column was tested in the chimera ELISA. Region-specific titers (% of total) were calculated as the ratio of end-point titers against a 3D7 chimera relative to the end-point titer against 3D7 AMA1 protein. (TIF) [file ppat.1003840.s008.tif]

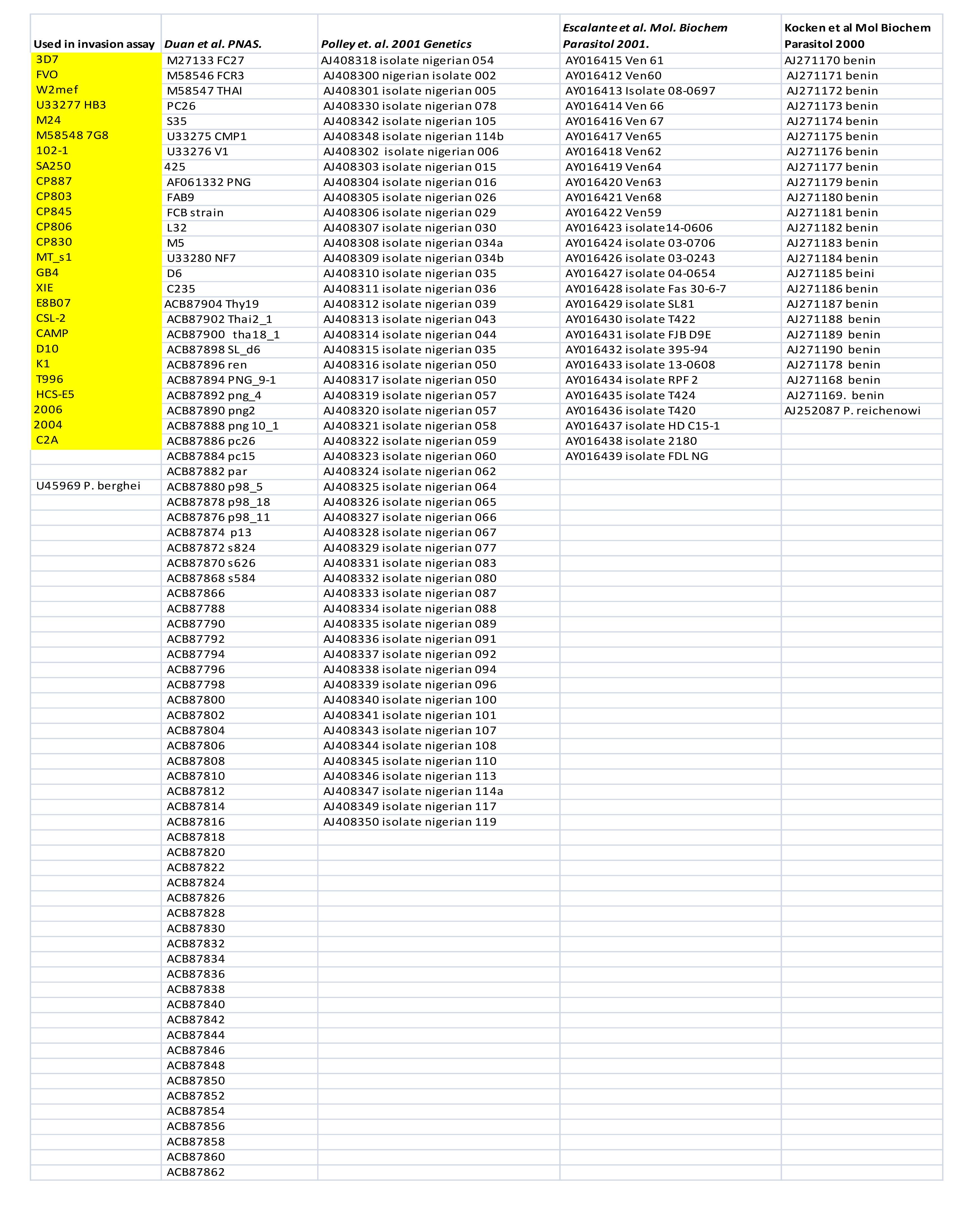

Supplement: Table S1 — The list of 201 isolates whose AMA1 sequences were used to create the dendrogram in Fig. 1A . The strains highlighted in yellow were tested in invasion inhibition assays and found to be susceptible to QV antibodies. AMA1 field isolate sequences were obtained from Genbank [35], [43], [87], [88] and lab isolates sequences were obtained from either Genbank or the source laboratory. (TIF) [file ppat.1003840.s009.tif]
