# Supplementary material for: Overcoming Antigenic Diversity by Enhancing the Immunogenicity of Conserved Epitopes on the Malaria Vaccine Candidate Apical Membrane Antigen-1
Source: PLoS Pathog. 2013 Dec 26;9(12):e1003840. doi: 10.1371/journal.ppat.1003840 (PMC3873463; doi:10.1371/journal.ppat.1003840)
Supplement: Table S2 — Sequence of protein chimeras. An alignment of P. berghei ANKA strain AMA1 is shown along with the residues that were switched to P. falciparum 3D7 sequence (boxed in gray). The boundaries of loops and domains are shown. (DOCX) [file ppat.1003840.s010.docx]

10 20 30 40 50 60

123456789012345678901234567890123456789012345678901234567890

P.berghei ANKA MKEIYYILILCSIYLINLSNCS---------------------------------EGPNN 27

P.falciparum 3D7 MRKLYCVLLLSAFEFTYMINFGRGQNYWEHPYQNSDVYRPINEHREHPKEYEYPLHQEHT 60

POLY EGPNN

CONS EGPNN

CryD1 EGPNN

CryD2 EGPNN

CryD3 EGPNN

HT EGPNN

D2+1e EGPNN

|-Lin D1

70 80 90 100 110 120

123456789012345678901234567890123456789012345678901234567890

P.berghei ANKA VISENGHIN----------------------YDMIQKENTERSTKLINPWEKYMEKYDIE 65

P.falciparum 3D7 YQQEDSGEDENTLQHAYPIDHEGAEPAPQEQNLFSSIEIVERSNYMGNPWTEYMAKYDIE 120

POLY VISENGHIN----------------------YDMIQKENTERSTKLINPWEKYMEKYDIE

CONS VISENGHIN----------------------YDMISIEIVERSNYMGNPWTEYMAKYDIE

CryD1 VISENGHIN----------------------YDMIQKENTERSTKLINPWEKYMEKYDIE

CryD2 VISENGHIN----------------------YDMIQKENTERSTKLINPWTEYMAKYDIE

CryD3 VISENGHIN----------------------YDMISIEIVERSNYMGNPWTEYMAKYDIE

HT VISENGHIN----------------------YDMIQKENTERSTKLINPWEKYMEKYDIE

D2+1e VISENGHIN----------------------YDMIQKENTERSTKLINPWEKYMEKYDIE

|-1a-| |-1b--|

130 140 150 160 170

12345678901234567890123456789012345678901234567890123456

P.berghei ANKA KMHGSGIRVDLGEDARVENRDYRIPSGKCPVIGKGITIQNSEVSFLTPVATGDQSV 121

P.falciparum 3D7 EVHGSGIRVDLGEDAEVAGTQYRLPSGKCPVFGKGIIIENSNTTFLTPVATGNQYL 176

POLY KMHGSGIRVDLGEDARVENRDYRIPSGKCPVFGKGITIQNSNTTFLTPVATGNQYV

CONS EVHGSGIRVDLGEDAEVAGTQYRLPSGKCPVIGKGIIIENSEVSFLTPVATGDQSL

CryD1 KMHGSGIRVDLGEDAEVAGTQYRLPSGKCPVFGKGIIIENSNTTFLTPVATGNQYL

CryD2 EVHGSGIRVDLGEDARVENRDYRIPSGKCPVFGKGITIQNSEVSFLTPVATGDQSV

CryD3 KMHGSGIRVDLGEDARVENRDYRIPSGKCPVIGKGITIQNSEVSFLTPVATGDQSV

HT KMHGSGIRVDLGEDAEVAGTQYRLPSGKCPVFGKGIIIENSNTTFLTPVATGNQYL

D2+1e KMHGSGIRVDLGEDARVENRDYRIPSGKCPVIGKGITIQNSEVSFLTPVATGDQSV

|-1c-| |----1d/C1L---|

180 190 200 210 220

7890123456789012345678901234567890123456789012

P.berghei ANKA RSGGLALPKTDVHLSPITIDNLKTMYKEHPEIVKLNNMSLCAKHTS 167

P.falciparum 3D7 KDGGFAFPPTEPLMSPMTLDEMRHFYKDNKYVKNLDELTLCSRHAG 222

POLY KDGGLALPPTEPLLSPMTLDELRHFYKDNKYIKNLDELSLCAKHTS

CONS RSGGFAFPKTDVHMSPITIDNMKTMYKEHPEVVKLNNMTLCSRHAG

CryD1 KDGGFAFPPTEPLMSPMTLDEMRHFYKDNKYVKNLDELTLCSRHAG

CryD2 RSGGLALPKTDVHLSPITIDNLKTMYKEHPEIVKLNELSLCAKHTS

CryD3 RSGGLALPKTDVHLSPITIDNLKTMYKEHPEIVKLNNMSLCAKHTS

HT KDGGFAFPPTEPLMSPMTLDEMRHFYKDNKYVKNLDNMTLCSRHAG

D2+1e RSGGLALPKTDVHLSPITIDNLKTMYKEHPEIVKLNNMSLCAKHTS

|----1e---| |----1f---|

230 240 250 260 270

3456789012345678901234567890123456789012345678901234567

P.berghei ANKA FYVPGNNANSAYRHPAVYDKSNSTCYMLYVAAQENMGPRYCSNNANNDNQPFCFT 222

P.falciparum 3D7 NMIPDNDKNSNYKYPAVYDDKDKKCHILYIAAQENNGPRYCNKDESKRNSMFCFR 277

POLY FYVPGNNANSAYRHPAVYDDKDKKCHMLYVAAQENMGPRYCSNNESKRNQPFCFT

CONS NMIPDNDKNSNYKYPAVYDKSNSTCYILYIAAQENNGPRYCNKDANNDNSMFCFR

CryD1 NMIPDNDKNSNYKYPAVYDDKDKKCHILYIAAQENNGPRYCNKDESKRNSMFCFR

CryD2 FYVPGNNANSAYRHPAVYDDSNKTCYMLYVAAQENMGPRYCSNNANNDNQPFCFR

CryD3 FYVPGNNANSAYRHPAVYDKSNSTCYMLYVAAQENMGPRYCSNNANNDNQPFCFT

HT NMIPDNDKNSNYKYPAVYDKSNSKCHILYIAAQENNGPRYCNKDESKRNSMFCFR

D2+1e NMIPDNDKNSNYKYPAVYDKSNSTCYMLYVAAQENMGPRYCSNNANNDNQPFCFT

|-Lin D2

280 290 300 310 320 330

89012345678901234567890123456789012345678901234567890123

P.berghei ANKA PEKIEKYKNLSYLTKNLRDDWETSCPNKSIKNAKFGIWVDGYCKDYQKHTVHDSDS 278

P.falciparum 3D7 PAKDISFQNYTYLSKNVVDNWEKVCPRKNLQNAKFGLWVDGNCEDIPHVNEFPAID 333

POLY PEKIISFQNYTYLTKNLRDDWETSCPRKNIQNAKFGIWVDGYCKDYQKHTVHDSID

CONS PEKDEKYKNLSYLSKNVVDNWEKVCPNKSLKNAKFGLWVDGNCEDIPHVNEFPADS

CryD1 PAKDISFQNYTYLSKNVVDNWEKVCPRKSIKNAKFGIWVDGYCKDYQKHTVHDSDS

CryD2 PAKDIKYQNLTYLTKNLVDNWEKVCPRKNLQNAKFGLWVDGYCKDIPHVNEFPAID

CryD3 PEKIEKYKNLSYLTKNLRDDWETSCPNKSIQNAKFGLWVDGNCEDIPHHTVHDSDS

HT PEKIEKYKNLSYLSKNVRDDWETSCPNKSIKNAKFGIWVDGYCKDYQKHTVHDSDS

D2+1e PEKIEKYKNLSYLTKNLRDDWETSCPNKSIKNAKFGIWVDGYCKDYQKHTVHDSDS

|-------------------loop 2--------------------|

340 350 360 370 380 390

4567890123456789012345678901234567890123456789012345678901

P.berghei ANKA LLKCNQIIFNESASDQPKQYEKHLEDTTKFRQGVAERNGKLIGEALLPIGSYKSDQIKSH 338

P.falciparum 3D7 LFECNKLVFELSASDQPKQYEQHLTDYEKIKEGFKNKNASMIKSAFLPTGAFKADRYKSH 393

POLY LFKCNQIIFNESASDQPKQYEKHLEDTTKFRQGVAERNGKLIGEALLPIGSYKSDQIKSH

CONS LLECNKLVFELSASDQPKQYEQHLTDYEKIKEGFKNKNASMIKSAFLPTGAFKADRYKSH

CryD1 LFECNQIIFEESASDQPKQYEQHLTDYEKIKEGFKNKNASMIKSAFLPTGAFKADRYKSH

CryD2 LFECNKLVFELSASDQPKQYEKHLEDTTKFRQGVAERNGKLIGEALLPIGSYKSDQIKSH

CryD3 LLKCNQIIFNESASDQPKQYEKHLEDTTKFRQGVAERNGKLIGEALLPIGSYKSDQIKSH

HT LLKCNQIIFNESASDQPKQYEQHLTDYEKIKEGFKNKNASMIKSAFLPTGAFKADRYKSH

D2+1e LLKCNQIIFNESASDQPKQYEQHLTDYEKIKEGFKNKNASMIKSAFLPTGAFKADRYKSH

|-Lin D3

400 410 420 430 440

45678901234567890123456789012345678901234567890123456789

P.berghei ANKA GRGYNWGNYDSQNKKCYIFETKPTCLINDRNFIATTALSSTEEFEEQFPCDIYKNK 394

P.falciparum 3D7 GKGYNWGNYNTETQKCEIFNVKPTCLINNSSYIATTALSHPIEVENNFPCSLYKDE 449

POLY GRGYNWGNYDTENQKCYIFETKPTCLINDSSFIATTALSHPIEVENNFPCSIYKDK

CONS GKGYNWGNYNSQTKKCEIFNVKPTCLINNRNYIATTALSSTEEFEEQFPCDLYKNE

CryD1 GRGYNWGNYDSQNKKCYIFETKPTCLINDRNFIATTALSHTIEFEEQFPCDIYKNK

CryD2 GKGYNWGNYNTETQKCEIFNVKPTCLINNSSYIATTALSHPIEVENNFPCDIYKNK

CryD3 GRGYNWGNYDSENKKCYIFETKPTCLINNSSFIATTALSSTEEFENNFPCSLYKDE

HT GRGYNWGNYDSQNKKCYIFETKPTCLINDRNFIATTALSSTEEFEEQFPCDIYKNK

D2+1e GRGYNWGNYDSQNKKCYIFETKPTCLINDRNFIATTALSSTEEFEEQFPCDIYKNK

|----loop 3------|

450 460 470 480 490 500

01234567890123456789012345678901234567890123456789012345678

P.berghei ANKA INEEIKVLNKNISNGN------NSIEFPRIFISTDKNSLNCPCEPTQLTESSCNFYVCN 447

P.falciparum 3D7 IMKEIERESKRIKLNDNDDEGNKKIIAPRIFISDDKDSLKCPCDPEMVSNSTCRFFVCK 508

POLY IMEEIKVLNKNISNGN------NSIEFPRIFISDDKDSLKCPCDPEMLSNSTCRFFVCN

CONS INKEIERESKRIKLNDNDDEGNKKIIAPRIFISTDKNSLNCPCEPTQVTESSCNFYVCK

CryD1 INEEIKVLNKNISNGN------NSIEFPRIFISTDKNSLNCPCEPTQLTESSCNFYVCN

CryD2 INEEIKVLNKNISNGN------NSIEFPRIFISTDKNSLNCPCEPTQLTNSTCNFYVCN

CryD3 IMKEIERESKRIKLNDNDDEGNKKIIAPRIFISDDKDSLKCPCDPEMVSNSTCRFFVCK

HT INEEIKVLNKNISNGN------NSIEFPRIFISTDKNSLNCPCEPTQLTESSCNFYVCN

D2+1e INEEIKVLNKNISNGN------NSIEFPRIFISTDKNSLNCPCEPTQLTESSCNFYVCN

|---transmembrane---|

510 520 530 540 550 560

901234567890123456789012345678901234567890123456789012345678

P.berghei ANKA CVEKRQYIAENNDVEIKEEFRSEYESPSN-----QRVIVIIIFICVGIILVILLVGYFFK 502

P.falciparum 3D7 CVERRAEVTSNNEVVVKEEYKDEYADIPEHKPTYDKMKIIIASSAAVAVLATILMVYLYK 568

POLY CVEKRQYIAENNDVEIKEEFRSEYESPSN

CONS CVERRAEVTSNNEVVVKEEYKDEYADIPE

CryD1 CVEKRQYIAENNDVEIKEEFRSEYESPSN

CryD2 CVEKRQYVAENNDVEIKEEFRSEYESPSN

CryD3 CVERRAEVTSNNEVVVKEEYKDEYADIPE

HT CVEKRQYIAENNDVEIKEEFRSEYESPSN

D2+1e CVEKRQYIAENNDVEIKEEFRSEYESPSN
